# Neuroimaging Findings in Adolescents and Young Adults with Anorexia Nervosa: A Systematic Review

**DOI:** 10.3390/children8020137

**Published:** 2021-02-12

**Authors:** Kalliopi Kappou, Myrto Ntougia, Aikaterini Kourtesi, Eleni Panagouli, Elpis Vlachopapadopoulou, Stefanos Michalacos, Fragiskos Gonidakis, Georgios Mastorakos, Theodora Psaltopoulou, Maria Tsolia, Flora Bacopoulou, Theodoros N. Sergentanis, Artemis Tsitsika

**Affiliations:** 1 MSc “Strategies of Developmental and Adolescent Health”, 2nd Department of Pediatrics, “P. & A. Kyriakou” Children’s Hospital, School of Medicine, National and Kapodistrian University of Athens, 115 27 Athens, Greece; kappouk5@gmail.com (K.K.); myntou@gmail.com (M.N.); kourtesikaterina@yahoo.com (A.K.); elenpana@med.uoa.gr (E.P.); tpsaltop@med.uoa.gr (T.P.); mariantsolia@gmail.com (M.T.); tsergentanis@yahoo.gr (T.N.S.); 2 Department of Endocrinology-Growth and Development, “P. & A. Kyriakou” Children’s Hospital, 115 27 Athens, Greece; elpis.vl@gmail.com (E.V.); stmichalakos@gmail.com (S.M.); 3 First Department of Psychiatry, Medical School, National and Kapodistrian University of Athens, Eginition Hospital, 115 28 Athens, Greece; frgonid@med.uoa.gr; 4 Unit of Endocrinology, Diabetes Mellitus and Metabolism, Aretaieion Hospital, School of Medicine, National and Kapodistrian University of Athens, 115 28 Athens, Greece; gmastorak@med.uoa.gr; 5 Department of Clinical Therapeutics, “Alexandra” Hospital, School of Medicine, National and Kapodistrian University of Athens, 115 28 Athens, Greece; 6 Center for Adolescent Medicine and UNESCO Chair Adolescent Health Care, First Department of Pediatrics, “Agia Sophia” Children’s Hospital, School of Medicine, National and Kapodistrian University of Athens, 115 27 Athens, Greece; bacopouf@hotmail.com

**Keywords:** anorexia nervosa, neuroimaging, magnetic resonance imaging, diffusion tensor imaging, single photon emission computed tomography, magnetic resonance spectroscopy

## Abstract

Background: Anorexia nervosa (AN) is a serious, multifactorial mental disorder affecting predominantly young females. This systematic review examines neuroimaging findings in adolescents and young adults up to 24 years old, in order to explore alterations associated with disease pathophysiology. Methods: Eligible studies on structural and functional brain neuroimaging were sought systematically in PubMed, CENTRAL and EMBASE databases up to 5 October 2020. Results: Thirty-three studies were included, investigating a total of 587 patients with a current diagnosis of AN and 663 healthy controls (HC). Global and regional grey matter (GM) volume reduction as well as white matter (WM) microstructure alterations were detected. The mainly affected regions were the prefrontal, parietal and temporal cortex, hippocampus, amygdala, insula, thalamus and cerebellum as well as various WM tracts such as corona radiata and superior longitudinal fasciculus (SLF). Regarding functional imaging, alterations were pointed out in large-scale brain networks, such as default mode network (DMN), executive control network (ECN) and salience network (SN). Most findings appear to reverse after weight restoration. Specific limitations of neuroimaging studies in still developing individuals are also discussed. Conclusions: Structural and functional alterations are present in the early course of the disease, most of them being partially or totally reversible. Nonetheless, neuroimaging findings have been open to many biological interpretations. Thus, more studies are needed to clarify their clinical significance.

## 1. Introduction

Anorexia nervosa (AN) is a serious mental disorder affecting predominantly adolescent girls and young adult women. Although relatively rare, it is the third most common chronic disease during adolescence [1,2]. It is characterized by a significantly low body weight, an intense fear of gaining weight, a disturbed perception of one’s body image and a persistent lack of recognition of the seriousness of the condition [3]. Patients display marked treatment resistance or no response to treatment, frequent medical complications and a substantial risk of death. [4] The two designated subtypes of the disease are the restricting type (R—AN), which describes presentations in which weight loss is accomplished primarily through food restriction and/or excessive exercise and the binge eating/purging type (BP—AN), which is characterized by recurrent episodes of binge eating or purging behavior. The restricting subtype is associated with an earlier age of onset, a better prognosis, and a greater likelihood of crossover to the other subtype [3].

Coexisting psychiatric disorders include bipolar, depressive and anxiety disorders, as well as obsessive and compulsive disorder (OCD) (especially among those with the restrictive subtype) and alcohol and other substance use disorder (especially among those with the binge/purging subtype) [3]. The underlying mechanisms that drive anorectic patients to deprive themselves of food while being hungry and emaciated are not yet fully understood. Current knowledge suggests that the disease is an interface between genetic and biological predispositions, environmental and sociocultural influences, and psychological traits [5].

Compared to other mental health disorders, the number of neuroimaging studies in AN is relatively small, although the literature is rapidly growing. Structural magnetic resonance imaging (MRI) has been long used to investigate volumetric differences between AN patients and healthy controls (HC). The first systematic review of structural MRI studies in AN patients was that by Van den Eynde et al. who did not find clear evidence for global grey matter (GM) and white matter (WM) volume reductions, and unraveled only preliminary findings for regionally reduced GM [6]. Systematic reviews and meta-analyses that followed have reported significant global reductions in GM and WM volumes as well as significant increase in cerebrospinal fluid (CSF) [7,8,9]. In a recent meta-analysis, adolescent patients showed greater reduction in GM volume than adults (7.6% vs. 3.7%) [10]. Similarly, WM was also significantly reduced in both adolescent and adult patients, on average 3.2% and 2.2% respectively, while CSF was conversely increased by 15%. These findings seem to normalize after long-term recovery, especially for the adult population, while for adolescents data are scarce [7,10]. Regional volume decreases have also been detected, more pronounced in the cingulate cortex, the supplementary motor area (SMA) and the amygdala [11]. On the other hand, studies in adult patients have demonstrated increased GM volumes in the insula and orbitofrontal cortex (OFC) [12,13,14].

While MRI identifies volumetric changes, magnetic resonance spectroscopy (MRS), provides information about the metabolism of brain tissues. Proton-MRS or Phosphorus-MRS offers various data regarding membrane composition and functionality, neuronal consistence and glial cells integrity, thrοugh the measurement of various metabolites such as choline (Cho) and total choline-containing metabolites (tCho), glutamate/glutamine (Glx), N-acetyl-aspartate (NAA), myo-Inositol (mI), total creatine compounds (tCr) and ethanolamine containing metabolites. In line with structural MRI studies, GM seems to be more affected than WM and these molecular changes could be identified before structural alterations become apparent, providing the benefit of early intervention [15,16].

In parallel with structural neuroimaging, researchers have further investigated functional brain processes to elucidate the causative underpinning of the disease. One of the most frequently used brain imaging techniques is functional magnetic resonance imaging (fMRI). Altered activation of the amygdala and insula has been consistently observed in patients with AN during passive viewing of visual food stimuli [17]. With regard to taste stimuli, most studies conclude to increased activation in reward-related regions [18,19]. In addition, fMRI studies utilizing body image related tasks have reported alterations of the precuneus, the inferior parietal lobe, the prefrontal cortex (PFC), the insula and the amygdala [20]. A recent systematic review of fMRI studies has provided evidence of impaired cognitive flexibility and social cognition skills in adolescent patients [21].

These published papers have reported considerable inconsistencies which could be attributed to differences in study design, selection of the stimuli or task and to the cognitive abilities of the participants. An approach to potentially overcome these inconsistencies is the investigation of resting-state functional connectivity (RSFC). With this technique potential biases stemming from the effort to perform a task are diminished, although unwanted thoughts, emotional status and ruminations could potentially affect RSFC [22]. The first RSFC study in AN adult patients was conducted by Cowdrey et al., who found increased RSFC between the default mode network (DMN) and the precuneus and the dorsolateral prefrontal cortex (dlPFC) when comparing patients with controls [23]. In a recent systematic review of fMRI studies, functional alterations were encountered in areas and networks related to the main symptoms of the disease, such as impaired cognitive control and body image disturbances [24]. These functional alterations are in line with data emerging from electroencephalography studies, showing decreased electrical activity in frontal and parietal-occipital regions during cognitive tasks. Likewise, altered electrical activity in fusiform gyrus and parahippocampal gyrus is demonstrated during resting-state paradigms [25].

On the other hand, fMRI is unable to identify and evaluate more precisely the functionality of brain neurotransmitters. For that purpose, single-photon emission computed tomography (SPECT) has been used. This three-dimensional nuclear medicine imaging technique provides information about regional cerebral blood flow (rCBF), showing alterations in perfusion and thus, evaluating brain functionality [26]. Moreover, by injecting radionuclides that selectively attach to receptors, specific information about neurotransmission can be provided. In particular, the serotonin neuronal system has been extensively studied, as it is believed to play a crucial role in the pathophysiology of AN, being involved in many cognitive features of patients and, therefore, providing possible therapy targets [26,27,28].

In recent years, the micro-architecture of WM axons connecting the abovementioned areas has been further explored by using diffusion tensor imaging (DTI). Four indices of diffusion are commonly used: fractional anisotropy (FA), mean diffusivity (MD), axial diffusivity (AD) and radial diffusivity (RD). Monzon et al. were the first to systematically review the limited at that time literature on DTI studies in AN [29]. They reported alterations in a range of WM structures of the limbic system as well as the fronto-occipital fiber tracts. In a more recent systematic review, Gaudio et al. concluded that patients with AN showed mainly WM microstructure abnormalities in thalamo-cortical tracts and occipital-parietal-temporal-frontal tracts [30]. The researchers were not able to draw a clear conclusion whether these alterations persist after recovery or not. To our knowledge, three meta-analyses have been published so far. In the first one, the quantitative voxel-based meta-analysis identified decreased FA in the posterior areas of the corpus callosum, the left superior longitudinal fasciculus (SLF) II and the left precentral gyrus as well as increased FA in the right corticospinal projections and lingual gyrus [31]. In the second one, Meneguzzo et al. identified two clusters of decreased FA, in the left corona radiata and in the left thalamus [32]. Finally, more recently, Zhang et al. analyzed DTI studies using tractbased spatial statistics (TBSS) and reported lower FA in the corpus callosum and the cingulum [33].

The aim of this paper is to systematically review current literature concerning structural and functional brain alterations in patients with AN, focusing on adolescents and young adults. Building on previous findings, our principal objective is to identify the main areas, networks and circuits that are vulnerable to the effects of the disease, while making an effort to disambiguate between effects of starvation and alterations possibly contributing to the pathogenesis of the disease.

### The Adolescent Brain

AN has its peak incidence between 13–18 years old [5]. Neuroimaging studies in adolescent patients at the early stages of the disease can provide the clinicians with valuable information with regard to early biomarkers before the effects of malnutrition become apparent. Nonetheless, the interpretation of findings is particularly challenging, as adolescence is a period of profound morphological and functional changes. In fact, adolescence is a very active period regarding neurodevelopment. A hallmark of the brain transformations is synaptic pruning, a highly specific process that has been speculated to help with the “rewiring” of brain connections into adult-typical patterns [34]. On the other hand, myelin production escalates during adolescence, leading to acceleration of speed and efficiency of information flow across distant brain regions [35]. In specific areas such as the PFC, myelin continues to increase until early adulthood, delaying maturation [36]. The combination of dendritic pruning and increased myelination of WM tracts results in the developmental “thinning” of the neocortex, a decline in thickness of outer layers of the brain that play an important role in high order functions [34]. Animal studies have shown that the abovementioned changes are critically driven by sex hormones [37]. Functional brain organization undergoes significant changes as well, shifting from a local connectivity pattern to a more distributed architecture [38,39]. In addition, the interconnectivity of core neurocognitive networks continues to change throughout adolescence, becoming other stronger or weaker. The net result of these developmental changes likely is the attainment of mature functional networks, optimally capable of supporting cognitive and behavioral demands [40]. Summarizing, we could suppose that the ongoing developing brain may be substantially vulnerable to the effects of starvation, which could disrupt the normal development. Understanding the normal developmental processes is critical for the interpretation of alterations observed in adolescent neuroimaging.

## 2. Materials and Methods

### 2.1. Search Strategy Criteria

The present systematic review was performed in accordance with the Preferred Reporting Items for Systematic Reviews and Meta-Analyses (PRISMA) guidelines [1]. A systematic and comprehensive search of PubMed, EMBASE and CENTRAL was carried out for papers published between database inception and October 2020. We used the following search algorithm: brain AND (“anorexia nervosa”) AND (“computed tomography” OR CT OR SPECT OR “SPECT-CT” OR “magnetic resonance imaging” OR MRI OR “functional MRI” OR “functional magnetic resonance tomography”). End-of-search date was set at 5 October 2020. Further article identification through reference lists of full text examined papers completed the research.

### 2.2. Selection Criteria

The eligibility criteria were based on the PICOS (Participants, Intervention, Comparison, Outcomes, Study design) acronym. To be included in the review, studies had to fulfill the following criteria: (i). written in English language; (ii). investigated participants aged between 10 and 24 years old with a current diagnosis of AN; (iii). were of case-control, cross-sectional or longitudinal design; (iv). excluded patients with a comorbid psychiatric disorder other than depression, anxious disorder or OCD; (v). excluded participants taking psychotropic medications other than antidepressants or anti-anxiety medications. Regarding fMRI studies, only resting-state fMRI studies were included, while task-based or stimuli-based fMRI papers were excluded due to space limitations. Due to the lack of sufficient papers using the same methodological approach, a meta-analysis was not performed. 

### 2.3. Quality Assessment and Data Extraction

Two authors (KPK and MSN) independently screened titles and abstracts from retrieved papers and analyzed full-text articles that met the eligibility criteria. Disagreements were resolved through consensus. The quality of the final studies was assessed by using the Newcastle–Ottawa scale. Two reviewers independently performed data extraction as follows: study design, demographic information (age, gender), sample size, age (mean age, age range), Body Mass Index (mean BMI), AN subtype, criteria for diagnosis, illness duration, co-morbidity and medication, scanning method and data analysis method. For DTI studies in particular, hydration status before neuroimaging was extracted as well. 

### 2.4. Search Results and Selection of Studies

**Figure 1:**
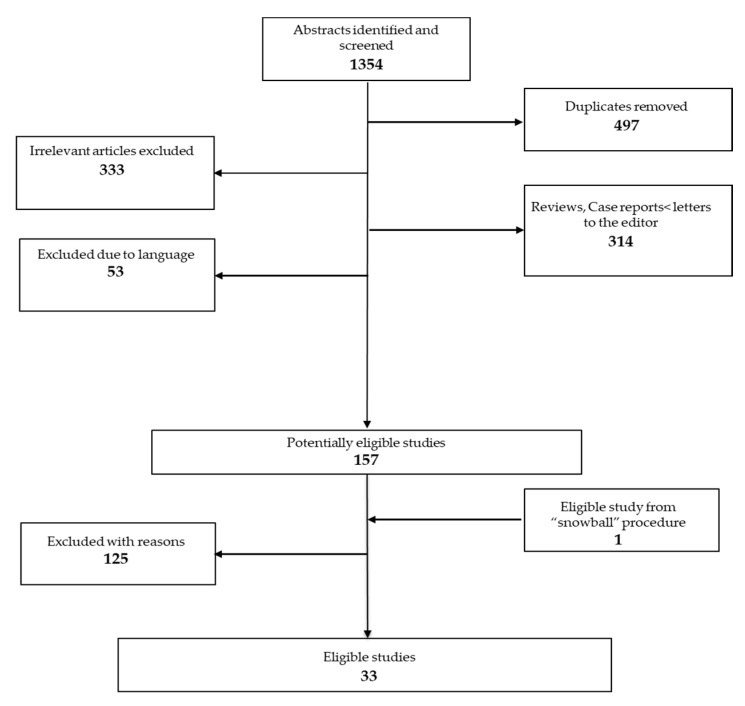
PRISMA flow chart.

The search strategy yielded 857articles, 33 of which met the inclusion criteria. The PRISMA flow diagram (Figure 1) shows selection and exclusion of studies.

### 2.5. Compliance with Ethics Guidelines

This article consists a review of previously conducted studies, which complies with the PRISMA guidelines [41].

## 3. Results

### 3.1. Study Characteristics

Selection of studies is presented in the flow chart (Figure 1); full-text studies excluded due to various reasons (*n* = 125) are presented in Supplemental Table S1. Overall, the 33 eligible studies included 587 participants with a current diagnosis of AN and 663 HC. Of the 33 studies, 31 were cross-sectional (9 also had a longitudinal follow-up) and 2 were longitudinal studies. Eighteen studies were conducted in a sample of exclusively adolescent patients for a total of 310 patients and 307 controls, while the remaining included both adolescents and young adults, for a total of 277 patients and 356 controls. Twenty-three studies examined structural imaging. Thirteen studies used structural MRI, 7 used DTI (2 of which provided also structural MRI data) and 3 used MRS. Their description is summarized in Table 1 and Table 2. Ten studies examined functional imaging. Six studies performed resting-state fMRI (3 of which performed also structural MRI) and the remaining 4 used SPECT. Their characteristics are presented in Table 3. Risk of bias assessment is presented in Supplemental Table S2a,b.

### 3.2. Results of Individual Studies

#### 3.2.1. Results of Structural Imaging Studies

##### MRI Studies

Thirteen studies using MRI scan to investigate structural abnormalities in participants with AN were systematically reviewed in the present study. Overall, the studies included 247 individuals with a current diagnosis of AN (148 with the restrictive subtype of the disease, 30 with the binge/purging subtype, 22 with atypical AN and 47 with unspecified AN) and 298 HC, mostly females (2 males only). All of the studies were cross-sectional, while 7 included also a longitudinal follow-up. Eight of the included studies were conducted in a sample of exclusively adolescent patients. Nine patients in total had a co-morbid disorder, either depression or anxious disorder and were under medication. The research groups used different methodological approaches to analyze their data. In detail, four studies used VBM, two used SBM and two were region-of-interest studies (ROIs). Table 2 presents scanning methods, main findings and clinical interpretations.

Overall, the majority of studies reported volumetric differences between AN patients and HC. In detail, total GM seemed to be predominantly affected. Significant global reduction of GM volume was reported in seven studies. Region-specific changes in GM were also identified. Specifically, local decreases in GM volume were detected in the parietal and temporal lobes, bilateral frontal gyrus, dorsolateral and medial prefrontal cortex, insular cortex, cerebellum and mesencephalon [29,42,43,44,45]. Apart from GM volume, cortical thickness was found also to be reduced either globally (except for the temporal poles and the entorhinal cortex) or regionally in the left precuneus [44,46]. Along with cortical GM reduction, ten studies *reported* regional volumetric differences in the GM of various subcortical areas and brain structures. In particular, reduced GM volume was reported in the amygdala, hippocampus and cingulate gyrus [29,44,47]. Additionally, one study investigated hippocampal subfields and found all volumes but one to be significantly reduced [47]. Similarly, apart from hippocampus and amygdala, GM volume was found also reduced in other subcortical nuclei, such as thalamus, nucleus accumbens and putamen [44,46,48]. Nonetheless, two articles presented no differences in caudate nucleus [44,46]. In discordance with findings concerning GM, WM volume appeared to be considerably less affected. Only one out of eleven studies identified significant reduction of global WM volume [49]. Regarding total brain volume, only one research group reported a significant reduction [47], while increased CSF volume was found in five studies. In seven studies, participants were re-examined with a second MRI after partial or full weight restoration. Apart from one study which examined patients after 2 years of treatment, the remaining had a relatively short follow up period ranging from 12 to 14 months (mean = 7 months). Significant total GM volume increase and normalization of ventricular enlargement was reported in six studies [42,45,48,49,50,51,52], while in one study enlargement remained [53]. Significant regional volume reduction was reported in ACC, temporal poles and entorhinal cortex, caudate nucleus, mesencephalon and hippocampus [45,48,52]. Along with brain morphology normalization, improvement in disease symptoms was reported as well [52].

##### MRS Studies

Three cross-sectional studies using MRS were systematically reviewed in the present paper. One included also a longitudinal follow-up. In total, 42 female and one male patient with a current diagnosis of AN were included (36 R-AN and 7 BP-AN) and compared with 58 HC. All patients were adolescents. Only one patient had depression and was under antidepressant therapy. Demographics and neuroimaging findings are shown in Table 1 and Table 2 respectively.

The researchers investigated the metabolism of both GM and WM. In detail, in patients with acute AN significantly higher concentrations of tCho, tCr and Glx were found, while low levels of NAA, Glx, and mI were detected in the frontal cortex, with a tendency to normalize after weight restoration [15,54]. Finally, using metabolite ratios in order to evaluate alterations, a significantly higher Cho-Cr ratio and lower NAA-Cho ratio were pointed out in the WM of the parietal-occipital region of patients [55].

##### DTI Studies

Seven DTI studies in participants with AN were systematically reviewed in the present paper. Overall, the studies included 175 individuals with a current diagnosis of AN (109 with the restrictive subtype of the disease, 6 with the binge/purging subtype, 35 with unspecified AN and 25 with atypical AN) and 209 HC, all females. Table 1 reports the sample characteristics. All studies were cross-sectional while two of them included also a longitudinal follow-up, after partial body weight restoration. Four of the included studies were conducted in a sample of exclusively adolescent patients, while the remaining included both adolescents and young adults. Three patients in total had a co-morbid disorder, either depression or anxiety disorder and were under medication. Two studies provided also volumetric data from structural MRI [56,57]. The research groups used different methodological approaches to analyze their data. In detail, two studies adopted VBM, three studies TBSS and two studies applied tractography. Table 2 presents scanning methods, main findings and clinical interpretations.

Overall, the majority of studies reported widespread alterations in diffusion parameters in several WM tracts. Only two studies did not detect any differences in the microstructure of WM comparing patients with controls [56,58]. Starting with association fibers, three studies reported WM abnormalities in the SLF, although findings were inconsistent [30,57,59]. Specifically, in the right SLF, FA was found decreased by Travis et al. and increased by Von Schwanenflug et al. [57,59]. Similarly, in the left SLF, FA was reported increased by Travis et al. and reduced by Gaudio et al., who additionally found decreased AD in the same WM tract [30,59]. Three studies pointed out WM alterations in the thalamic radiation [57,59,60]. In particular, FA was increased in the acute stage when compared with controls [59,60] but was also increased in patients having partially restored their body weight when compared with the acute stage of the disease [57], while no difference was encountered at baseline. In this patient group, Von Schwanenflug et al. found additionally higher FA in the fornix [57]. One more study reported results regarding the fornix. In particular, Travis et al. found decreased FA in this area [59]. Two studies reported WM alterations in the corona radiata [30,60]. Gaudio et al. showed decreased FA and AD in the left superior and anterior corona radiate [30]. On the other hand, Vogel et al. found increased FA in the bilateral superior corona radiata as well as the anterior and posterior limb of the internal capsule [60]. Following with commissural fibers, three studies highlighted WM alterations in the corpus callosum [57,59,60]. Again, findings were conflicting, with FA value being reported either increased or decreased. Moreover, Travis et al. estimated R1, a myelin index, which was measured decreased mainly in the body and splenium of the corpus callosum [59]. The same index was also reduced in corticospinal tracts. With regard to projection fibers, only one study reported reduced FA in the right corticospinal tract with weight gain, while no difference was detected in the acute stage compared to controls [57]. In contrast with the other research groups, Hu et al. localized their results in GM, reporting decreased FA in several cortical regions, mainly in the frontal lobe, the cingulum, the thalamus and the insula [61]. Finally, as already mentioned two studies did not find any differences between patients and HC, in any of the diffusion indices [56,58].

#### 3.2.2. Results of Functional Imaging Studies

##### fMRI Studies

Six resting-state fMRI studies in participants with a current diagnosis of AN were systematically reviewed in the present paper. Overall, the studies included 78 individuals with a current diagnosis of AN (76 with the restrictive subtype of the disease and 2 with the binge/purging subtype and 76 HC, all females. Table 3 reports the sample characteristics. All were case-control studies. Two of the included studies were conducted in a sample of exclusively adolescent patients, while the remaining included both adolescents and young adults. Only one patient in total had a co-morbid disorder and was under antidepressant medication. Three studies provided also volumetric data from structural MRI. The research groups used different methodological approaches to analyze their data, either whole brain approaches, or network based. Table 4 presents scanning methods, main findings and clinical interpretations.

Overall, all studies reported disturbed widespread functional connectivity alterations in several brain regions. The variety in the location of findings defies the strict categorization of results by area of interest. In detail, as already mentioned, two studies included only adolescent patients at the earliest stage of the disease. In the first one, Gaudio et al. identified eight widely accepted resting-state networks [62] and found decreased functional connectivity between the ECN and the ACC, which correlated positively with BMI [63]. In the second study in adolescents, Gaudio et al. reported decreased connectivity in a subnetwork involving the ACC, the paracentral lobule, the cerebellum, the insula, the orbitofrontal gyrus and the occipital gyrus (see Table 4 for details) [64]. Neither of the two studies found volumetric differences between patients and HC. Of the remaining four studies, one research group focused as well on resting-state functional networks. In particular, Bohem et al. reported increased functional connectivity between the angular gyrus and other parts of the FPN and also between the anterior insula and the DMN [65]. One study, that of Amianto et al. centered on the intrinsic connectivity of the cerebellum and found increased connectivity within the insula, vermis, temporal poles and PCC and decreased connectivity with the parietal lobes [66]. Cerebellar atrophy was an additional finding. Finally, two research groups pointed out decreased functional connectivity in the thalamo-insular network [67,68]. Furthermore, Geisler et al. highlighted an altered global network architecture [67].

##### SPECT Studies

Four articles performing SPECT scan in adolescents and young adults with acute AN met the inclusion criteria. A total of 44 patients were included (25 with R-AN, 9 with BP-AN and 10 unspecified) along with 19 HC, all females. Two studies were cross-sectional, one of which re-examined 12 patients after partial weight gain and two were longitudinal studies re-examining their patients (*n* = 18) after partial weight gain or normalization. One study was conducted in a sample of exclusively adolescent patients. None of the patients had any co-morbid disorders. Demographic data and clinical findings are presented in Table 3 and Table 4, respectively.

Hypoperfusion was reported in frontal, parietal, temporal, and occipital regions in acutely ill individuals compared to HC [69,70]. Decreased perfusion was positively correlated with BMI [69]. After partial recovery, blood perfusion showed a tendency to increase and reached almost normal levels. Conversely, elevated blood flow was reported in the thalamus, amygdala and hippocampus [69,70]. 

When examining patients after a BMI elevation by 50%, a significant increase in rCBF was reported in the right dlPFC, the medial parietal cortex including the precuneus and in the PCC. In contrast, a decrease in rCBF was found in the right putamen and a positive correlation was reported between rCBF in the right DLPFC and the interoceptive awareness score in patients before treatment, while after treatment no correlation was found [71]. After an increase in BMI by 25%, significantly increased rCBF was found in bilateral parietal lobes and right posterior cingulate gyrus and a positive correlation was reported between BMI and rCBF in the right thalamus, parietal lobe, and cerebellum [72].

## 4. Discussion

### 4.1. Discussion of Structural Imaging Studies

Starting with MRI, the most frequently used neuroimaging method, total and regional GM volume reduction appears to be the prominent finding, although some heterogeneity arises in terms of severity and localization of findings. This ‘pseudo-atrophy’ shows the tendency to reverse after weight gain, at least in non-chronic patients. In addition to global GM reduction, particular regions appear to be more susceptible to volume loss, such as the parietal cortex, precuneus, insula, thalamus, as well as limbic structures, such as the PFC, cingulate cortex, hippocampus and amygdala [9,10].

Although several pathophysiological mechanisms have been suggested, the exact etiology of GM atrophy remains unknown. It is widely believed to be the result of extreme malnutrition and not a predisposing factor to the disease. Patients with AN follow a restrictive diet pattern, excluding polyunsaturated fatty acids, proteins and neuroprotective nutrients, such as B-complex vitamins and antioxidants [14]. Polyunsaturated fatty acids are exclusively received through diet and are essential for neuronal membranes integrity and function. Their nutritional deficiency is associated with neuronal apoptosis and interruption of the normal cortex maturation during adolescence, which may explain the greater GM vulnerability during this period [73]. In addition, the decreased protein synthesis due to the extreme nutrient deprivation is thought to result in a reduced number of synaptic connections and delayed synaptogenesis [50]. Other published theories to explain atrophy are brain shrinkage due to dehydration and fluid shifting to extracellular space due to osmotic alterations [74]. However, in almost every included study, MRI imaging was conducted after the initial stabilization of patients and with albumin and electrolytes serum levels within the normal range. Thus, dehydration or osmotic alterations do not seem to adequately explain findings. Considering the hormonal status of the patients, elevated cortisol levels, which is a common finding, are significantly associated mainly with intraparenchymatic GM volume depletion [49]. The underlying mechanism is still unknown, but it is likely that cortisol may be responsible for alterations in protein catabolism [75]. Moreover, secondary insulin growth factor-1 (IGF-1) deficiency due to malnutrition can result in loss of oligodendrocyte proliferation and differentiation, inhibition of myelination and eventually brain volume reduction [76].

An interesting theory that has been proposed to explain the greater GM loss in adolescents, is that the disease seems to modify the normal process of brain maturation (pruning), leading to a more “ergonomic” cortical architecture in order to save energy and maintain an adequate network efficiency despite starvation and malnutrition. This theory may also explain the clinical observation that patients feel actually more alert when starving, at the early stages of the illness [77]. Interestingly, in adolescents with a recent diagnosis of atypical AN, no significant volumetric differences were detected, and no clinical correlations were found. It is worth mentioning though, that patients had a significantly lower BMI than HC and that 40% of them presented with secondary amenorrhea. It is therefore suggested, that a critical BMI limit exists, below which the loss of GM begins to become apparent [78]. This hypothesis is supported by a positive correlation between volumetric alterations and the weight loss rate, emphasizing the importance of early diagnosis and intervention [79]. Overall, the positive correlation between patient’s BMI (and not disease duration) and global GM volume depletion in combination with atrophy reversion after partial weight gain, lean towards the concept that atrophy is a temporary effect of semi-starvation and not a consequence of major cell apoptosis, at least in non-chronic patients [50,80].

As already mentioned, specific brain regions appear to be more affected in terms of GM loss, indicating either a higher vulnerability to the disease or a potential involvement in AN pathogenesis. In particular, the parietal lobe is reported by several research groups to be primarily affected [42,46,48]. This finding has been linked to the reported cognitive deficits in verbal memory and to the impairment of visuospatial and concentration ability of patients [9]. Additionally, parietal volume loss may contribute to size overestimation, an important element of disturbed body image. Interestingly, the distortion of self-body image in AN patients is quite similar to the distortion reported after right parietal lobe damage (e.g., stroke), indicating the existence of a cognitive, non-emotional component of body misperception [81]. Another prominent finding is the involvement of limbic structures. The hyperactive adolescent limbic system is believed to have its own special role in the neurobiology of AN [82]. Hippocampus and amygdala in particular, appear to be more susceptible to volume loss. Hippocampal atrophy is reported in several other serious psychiatric disorders, such as major depression [83]. Lower hippocampal volumes in these individuals appear to be a result of longer illness duration or greater number of episodes, instead of a premorbid vulnerability factor [84]. Excessive cortisol levels due to chronic stress have been proposed as the driving mechanism [85]. In AN, hypercortisolemia may serve as a compensatory mechanism, increasing gluconeogenesis and providing vital nutrients [86]. Nonetheless, patients often have co-morbid anxiety disorders, so stress can actually be a confounding factor [87]. This notion is supported by the partial increase in the hippocampal volume following the administration of antidepressants [88]. When comparing studies conducted in teens to those in adults, greater atrophy is shown in adolescent hippocampus [7,47,89]. This exceptional vulnerability to stress during this period may be the result of rapid brain development and increased plasticity, in combination with the existence of numerous stress-hormone receptors in this area [90].

Another limbic structure that seems to have a pivotal involvement in the disease symptomatology and potentially pathophysiology is the amygdala, the brain’s ‘’threat detector’’ [91]. Exaggerated activity of the amygdala is observed in healthy adolescents compared with adults [92]. This hyperactivity is argued to have a central role in AN [93]. Specifically, it has been suggested that a trait amygdala hypersensitivity exists for individuals who will develop AN contributing to the experience of emotion as overwhelming and aversive, and which may become further aggravated during adolescence corresponding with disorder onset [94]. In detail, AN patients, especially those with R-AN, experience food stimuli very aversively, displaying an increased reactivity of the right amygdala [93]. In line with this finding, fMRI studies report hyperactivity of the amygdala when patients are exposed to gustatory [19], or body-related paradigms [95,96], or even disease specific words [97]. These results are suggestive of a multimodal amygdalar reactivity independent of the sensory mode. Likewise, this amygdalar hyperactivity is elicited by disorder-unrelated (emotional) stimuli, reflecting a heightened negative emotional arousal [98]. It could be argued that AN patients are biased towards both emotional (disease-unrelated) and non-emotional (disease-related), a feature that persists following recovery [94,99]. Interestingly, increased activation of the amygdala during anticipation of food is reported also in siblings of AN patients, a finding that supports further the notion of amygdalar hypersensitivity as a premorbid biomarker [100]. Amygdala activation after gustatory stimulation may be the result of intense fear of weight gain [19]. It has been proposed that increased activity in the amygdala may lead to fearful emotional processing concerning body image issues, and in turn influences calorie intake and weight gain [97]. Reduced amygdala volume in AN patients has been associated with less body image uncertainty and reduced phobia scores, possibly contributing to the disease maintenance [101]. It is also worth mentioning that the amygdala, along with the insula and other limbic structures, is part of complex neural circuits related to emotional perception [102]. Thus, in the backdrop of an hyperactive amygdala sending high levels of negative emotional threat information, the insula is unable to integrate basic emotion detection [94]. The pivotal role of the insula in the disease is discussed further along with other functional alterations.

Apart from volumetric changes, alterations in GM metabolism have been studied as well with MRS imaging, although this is not a widely used technique across neuroimaging research of eating disorders. In general, the rarity of MRS data precludes definite conclusions.

MRI imaging has not revealed significant volumetric changes regarding WM. However, changes in the microarchitecture of WM became evident through DTI studies. Overall, multiple WM alterations have been demonstrated, with some degree of overlap across studies but also with a relative inconsistency regarding the location and direction of alterations. With regard to localization of findings, the microarchitecture of WM appears to be affected mainly in thalamo-cortical connections (corona radiata, thalamic radiation), in interhemispheric connections (corpus callosum), in tracts connecting cortical regions as well as in various regions of the limbic system such as the fornix, insula, cingulum and frontal areas [103]. The abovementioned WM tracts and GM areas are involved in somatosensory, emotional and reward processing, in high order cognitive functions and in the formation of body image perception. Therefore, WM alterations may have potential clinical implications related to the symptomatology of the disease. 

In detail, the corona radiata is a key WM structure of the DMN, connecting the cerebral cortex to the basal ganglia and the brain stem [104,105]. Disruption of WM could be related to cognitive and emotion regulation deficits in anorectic patients, as has already been demonstrated in individuals with bipolar depression [106]. The posterior thalamic radiation connects thalamus with the parietal and occipital lobe, regions that are anatomically and functionally linked to body image. It could be hypothesized that alterations in WM micro-architecture of thalamic radiation may correlate with distorted body image. However, further research is needed to verify this assumption. The corpus callosum is the principal WM fiber bundle of the brain, involved in motor, perceptual and cognitive functions. Alterations in WM could be related to reduced quantity and speed of information between these areas [33]. Furthermore, corpus callosum atrophy has been correlated with cognitive flexibility, another core feature of the disease [107]. Likewise, the SLF is a major intrahemispheric WM and a major link between the PFC and the parietal lobe concerning the perception of the visual space, providing a means by which the PFC can regulate the focusing on attention in different parts of space [108]. Altered SLF microstructure could give rise to body size misperception, by disrupting information flow across cortical regions implicated in visual attention, spatial perception, and body-specific processing [108,109,110]. Finally, the fornix is a major limbic structure which is involved in reward processing and feeding regulation [111]. Previous studies have reported consistently reduced FA in the fornix in AN linking this structure with disease symptomatology and potentially pathophysiology [111]. In our review, FA was found either decreased or increased [57,59]. It is worth mentioning that both research groups took into account that FA may be biased by ventricular enlargement which is often encountered in AN patients, due to the partial volume effect (PVE), which occurs when voxels contain heterogenous tissue types, i.e., WM tissue and CSF in the case of the fornix [112]. For that reason, the former estimated ventricular size prior to DTI scanning, while the latter considered ventricular volume as a covariate in their analysis. 

As already mentioned, there is a relative discrepancy concerning the localization of findings, which may be attributed to the different methodological approaches utilized for data analysis, (i.e., VBM, TBSS, tractography) leading researchers to focus on different brain regions. Given this variance, it is worth mentioning the study of Pfuhl et al. who found no differences between patients and controls [56]. The researchers applied a global probabilistic tractography, a different analysis that may not detect subtle or more localized alterations [57]. On the other hand, the inconsistency in terms of the direction of the alterations is quite impressive, if not unexpected. In particular, FA was reported either decreased or increased in the same regions of interest by different researchers, a finding that comes in contrast with previous findings in adults who have consistently reported decreased FA [113,114,115]. Increased FA is a finding encountered principally in adolescents, in line with previous studies [116]. This discrepancy was pointed out in the meta-analysis of Barona et al. as well [31]. It is a fact, that the interpretation of the DTI findings is challenging, as indices of diffusion are open to many biological interpretations. FA in particular, is a highly sensitive but non-specific biomarker of brain WM microstructure [117]. Decreased FA combined with increased MD is typically interpreted as disturbed WM integrity, whereas increased FA is thought to reflect increased myelination [118]. However, increased FA may not always be a desirable finding. For instance, a higher FA value in auditory fibers has been reported in patients with schizophrenia who suffer from hallucinations [119]. In addition, as was pointed out by Jones et al., FA may be affected by several other factors, such as larger axon diameter, lower fiber density, increased membrane permeability and reduced myelination [120]. FA values could additionally be affected by the reduced WM volume, a phenomenon probably attributed to the reduction in the number of supporting glial cells, in the size of neurons and glia cell bodies, or in altered protein synthesis that results in fewer and smaller dendrites and synaptic junctions [8]. A recent study in an animal model of AN identified strongly reduced astrocyte count and astrocyte volumes in the WM of the brain [121]. The reduction in the surrounding tissue could also be a consequence of dehydration [60]. WM with large axons, such as the corpus callosum and the corticospinal tracts have thicker myelin sheets and larger concentrations of myelin. Thus, they may be more vulnerable to the effects of starvation [122]. Another factor that should be taken into consideration for the interpretation of DTI findings is the phenomenon of crossing fibers. A voxel may be composed of fibers with different spatial orientation resulting in an increase in average FA, without reflecting changes in myelin structure [120]. Moreover, most studies focused on FA, which is the most commonly reported variable, and secondarily on MD. However, these may not be enough to characterize DTI changes. AD and RD are considered to be more specific to underlying biological processes, such as myelin and axonal changes [123].

The neuroimaging findings in adolescent patients are of special interest. The detection of site-specific WM alterations in young patients and at the earliest stages of the disease, as described in two studies, supports the hypothesis that these alterations may represent premorbid trait markers [30,59]. On the other hand, Vogel et al. reported widespread alterations, which could be hardly correlated with specific symptoms of the disease and which rapidly normalized with weight restoration [60]. Additionally, the researchers found a positive correlation between FA and the speed of weight loss. Taken together, these findings are against the aforementioned hypothesis. Interestingly, Olivo et al. did not detect any diffusivity abnormalities in adolescents with atypical AN, supporting the notion that undernutrition is the underlying mechanism of FA alterations [58]. This subgroup of patients is characterized by the typical features of AN but with a body weight within the normal limits. In an attempt to interpret the results, it could be speculated that the developing adolescent brain reacts to malnutrition in a different way compared to adults, in a way that even the minimum limitation of food intake could affect the WM development. Alternatively, the increase in FA, which is the prominent finding in adolescents, could reflect a compensatory mechanism to starvation before the long-lasting deprivation of food results in the reduction of FA in adulthood. One way or another, during adolescence, WM maturation is characterized by continual widespread changes of increased FA and increased MD in widespread areas of cerebral and cerebellar WM, prominently in the frontal lobes and association fibers that connect them to other parts of the brain. These changes are driven by reductions in both AD and RD [124]. AD reduces with age, probably as a result of increased numbers of brain fibers or increased axonal caliber and the growth of glial cells. RD also reduces with age as a result of increased myelin development in the majority of brain areas [124]. Thus, normal baseline and age-related RD and AD values should be taken into consideration when investigating pathological conditions in this age group. 

Overall, literature indicates that WM is affected in young patients with AN. However, the exact nature of WM alterations is unclear, and no safe conclusions can be drawn whether these alterations bear on disease pathophysiology or not. 

#### 4.1.2. Discussion of Functional Imaging Studies

Overall, the researchers reported an altered functional connectivity across various brain regions and large-scale resting-state networks. Despite the discrepancies among studies (in terms of data analysis method and distribution of findings), results indicate functional abnormalities across several areas and networks related to core features of AN such as cognitive inflexibility, disturbed body image and deficits in emotional processing and executive control. In particular, most of the abovementioned areas belong to either the limbic or reward system and additionally are considered to be part of the well identified resting-state networks [24].

One of these areas of special interest is the ACC, a region with multiple functions and several functional connections at rest. The ACC lies in the medial wall of each cerebral hemisphere and is connected to both the “emotional” limbic system and the “cognitive” prefrontal cortex [125]. It is also considered a part of the ECN [63]. It is involved in cognitive and sensorimotor functions as well as in affect-regulation, i.e., the ability to control and manage uncomfortable emotions [126,127]. Stimuli-based fMRI studies in healthy individuals have revealed the role of the ACC in the emotion-regulation process, through a generalized “top-down” control from the prefrontal cortex, which provides the capacity to regulate an over-activated emotional response from the limbic system [125,128]. On the other hand, fMRI studies in patients with psychiatric disorders have reported that both hyper- and hypo-activation of the ACC is involved in impaired emotion regulation characterizing depression, schizophrenia and posttraumatic stress disorder (PTSD) [129,130,131]. Likewise, altered functional connectivity of the ACC in AN could support the notion that emotion dysregulation is associated with the appearance, maintenance and outcome of the disease [132]. It is finally worth mentioning that functional alterations of the ACC were early recognized by SPECT imaging. Hypoperfusion of this region was a consistent finding, not completely normalizing after weight restoration. However, with the introduction of PET imaging to eating disorders research, further data from SPECT no longer exist.

Another area of interest is the insula. The insular cortex is implicated in an overwhelming variety of functions such as sensorimotor processing, emotional awareness, autonomical control, risk prediction, decision-making and complex social functions like empathy [133,134]. An additional key function of the insula is the integration of interoceptive information, i.e., internal physical sensations including pain, hunger and thirst [87]. The right insula specifically is involved in “self—recognition” [135]. MRI studies have revealed increased volume of the right insula in adolescent and adult patients with AN [116]. This finding has also been correlated with the rumination of being fat while actually being emaciated [136]. Furthermore, the anterior insula is the primary gustatory cortex. Along with the ACC and the OFC, the anterior insula codes the sensory-hedonic response to taste. Moreover, it may play a crucial role in linking sensory-hedonic experiences to the motivational component of reward, which urges an individual to approach food [87]. This potential contribution of the insula to eating behavior has been highlighted in functional neuroimaging. Previous fMRI studies in recovered AN patients have reported a reduced insula response to sweet taste when compared to controls [137,138]. Likewise, a study in participants with acute AN has revealed insula activation in response to drinking chocolate milk in controls but not in patients, in the satiety state [19]. In contrast, a neuroimaging study in healthy individuals pointed out that food deprivation, compared to the satiety state, produces greater insula activation [139]. Adding to these findings, altered functional connectivity at rest between the insula and various regions, as well as within the networks that pass through it, supports the hypothesis that the insula has a central role in the pathophysiology of AN [140]. Interestingly, Bohem et al. found a positive correlation of functional connectivity in the anterior insula with difficulties in interoceptive awareness [65]. These findings come in line with the suggestion that many of the symptoms, such as distorted body image, lack of recognition of the severity of the situation (due to inappropriate response to hunger) and diminished motivation to change could be related to disturbed interoceptive awareness [87].

The prominent finding of altered functional connectivity of the ACC and insula could be interpreted in the context of adolescent brain maturation. It has been suggested that ACC is a key neural substrate of adolescent neurodevelopment [141]. Critically, ACC connectivity undergoes tremendous reorganization during adolescence [142]. In detail, the rostral ACC becomes more strongly connected to the DMN, whereas dorsal ACC shows increasing connectivity with the SN [141]. These developmental changes may contribute to the appearance of AN psychopathology during adolescence, given that most mental disorders are currently considered neurodevelopmental. Likewise, widely accepted theories regarding neurocognitive development in adolescence emphasize the different developmental trajectories of subcortical motivational and cortical control regions [143]. Specifically, limbic regions involved in reward and affective processing mature earlier than PFC regions for the executive control of the behavior, thus creating an imbalance in decision making. The fact that insula serves as a key hub in the interface between emotional processing and executive control [144], brings forward this structure as a central component of adolescent physiological maturation and potentially phychopathology as well. Indeed, research indicates that the fronto-insular connectivity displays the most dramatic developmental effects during puberty [145]. Taken together, this notion opens a new research direction towards the prioritization of the neurodevelopment to understand vulnerability to disease state [146].

Apart from the abovementioned functions, insula appears to be a central hub of some large-scale resting-state networks. A few researchers focused on the study of these networks and highlighted the altered functional connectivity within and between them. In detail, researchers attempted to explore the functional interactions between three core resting state networks (RSNs), the DMN, the ECN and the SN. The DMN is a well-recognized network which encompasses the medial prefrontal cortex, the posterior cingulate cortex, the precuneus, the inferior parietal lobule and the lateral temporal cortex. It has been hypothesized to be active during rest and deactivated when specific goal-directed behavior is needed [147]. In particular, the DMN is the most active brain system when individuals are left to think to themselves undisturbed. It is involved in mental explorations including remembering the past, envisioning the future, considering the thoughts of other people and thinking about one’s self [148]. Dysfunction of the DMN has been related to Alzheimer’s disease, schizophrenia and autism and virtually to every major psychiatric disorder [149,150]. The ECN covers the dlPFC and the lateral posterior parietal cortex and is responsible for high level cognitive functions such as planning and decision making. ECN disruption is also widespread in most mental disorders [151]. The SN covers the dorsal ACC and the anterior insular cortex and is involved in detecting and filtering internal and external stimuli [151]. Bohem and colleagues attempted to interpret their results in the framework of the triple network model of psychopathology suggested by Menon [65,151]. According to this model, deficits in engagement and disengagement of these three core neurocognitive networks (ECN, DMN, SN) play a significant role in many psychiatric disorders. This model highlights the crucial role of the SN, with the anterior insula as its central hub, in initiating the switch from the DMN to the ECN, for the generation of appropriate behavioral responses to salient stimuli. In detail, the researchers reported increased connectivity between insula and DMN, a finding that is in line with this model representing a difficulty in disengagement from a self-focused state of mind, intensifying the ruminative preoccupation with body image and food. Likewise, in individuals suffering from major depression, increased activation of the DMN has been positively correlated with depressive ruminations [152]. A similar approach was adopted by Uniacke et al. [153]. In their longitudinal study, researchers found reduced SN-ECN connectivity which remained after weight normalization.

Researchers have queried the extent to which this multinetwork model gradually emerges from childhood [146]. Converging evidence suggest the strengthening of intra- and inter- network connectivity in adults compared to children, implying that significant sub-network reorganization takes place during adolescence [38,39,145]. Network maturation follows a hierarchical modularity, with those networks serving the most basic functions of the organism maturing the earliest [146]. This asynchrony in the timing of network developmental trajectories might result in greater vulnerability to mental disorders during adolescence [146], among those AN as well. This theory has been previously proposed for the greater vulnerability of adolescents to addictive behaviors [146]. Nonetheless, the complex and highly sophisticated methodology of these studies results in sparse data, especially in adolescent populations. Further research is needed therefore, to clarify the potential role of the triple network connectivity in the pathophysiology of the disease and to further investigate the complex inter-network relationships. 

Another brain area with a potentially pivotal role in the disease is the OFC. This subregion of the prefrontal cortex has a major role in regulating when to stop eating a particular food, by activating the phenomenon of *sensory specific satiety*, a decline in pleasantness of a food as it is eaten [154,155]. The median OFC has further been associated with food avoidance. Altered functional connectivity in this area comes in accordance with the previously reported reduced grey GM matter volume in adolescents and adults with AN, a finding which has been correlated with disturbed satiety regulation, a possible driving mechanism for restriction of food in anorectic patients [116].

The interpretation of findings raises again the question whether functional connectivity could be affected by undernutrition. For example, Amianto et al. reported GM volume reduction in the same regions where abnormal connectivity was detected [66]. On the other hand, functional alterations in adolescent cohorts were not related to volumetric differences, although a positive correlation between ACC connectivity and BMI was highlighted [63]. Thus, no definite conclusion can be drawn. An additional, open to interpretation, aspect of functional connectivity is the direction of the effect. Although it could be obviously hypothesized that increased connectivity is desirable, its clinical implication is difficult to be assessed. Finally, it is worth mentioning that only two studies reported correlations between functional alterations and core symptoms of the disease [63,65]. Relating encountered differences between groups to relevant clinical variables increases the reproducibility of the results and thus, is an advisable approach for every study [156].

#### 4.1.3. Overall, Synthesis and Limitations

This systematic review attempted a global approach to structural and functional alterations in the brain of youth patients with AN. Young adults were also included in this effort, as they share more common features with the teen population than with older adults, due to the ongoing brain neurodevelopment during the first years of adulthood.

Our findings are consistent with the current literature indicating widespread and regional GM volume reduction, WM microstructure disturbances and resting-state functional alterations. The heterogeneity of findings across all neuroimaging methods may be attributed to the different methodological approaches and the non-uniformity of cohorts regarding multiple clinical variables such as disease duration and severity. Alternatively, it may merely reflect the complexity of the disease. In fact, specific brain regions such as the insula, PFC, parietal cortex, as well as WM tracts and functional networks related to them appear to be consistently affected in young patients, suggesting their potential role in the disease pathophysiology. Typical findings in adult patients such as cerebellar atrophy are not consistently encountered in young individuals, suggesting associations with longer disease duration [12,157,158]. A prominence of limbic structures is also indicative of emotional and reward processing deficits being at the root of the disease. Of course, in the human brain, it is not always possible to ascribe a symptom to a single region. On the other hand, both structural and functional alterations are highly reversible after short weight restoration and long before the psychological recovery, pointing out malnutrition as the underlying causative mechanism, although data from longitudinal studies are limited. 

A principal limitation of our review is the exclusion of stimuli or task-based fMRI studies, due to space limitations and in order to limit heterogeneity related to study design oriented to specific tasks. However, this exclusion precluded us from addressing disturbances in neural circuitries involved in reward-processing, which may have a central role in AN according to current neurobiological models of the disease [87,159]. Additionally, the application of stringent criteria in an effort to eliminate potential confounders such as psychiatric comorbidity and medications may have led us to exclude studies with significant results and has resulted in a limited number of resting-state fMRI and DTI studies. Another noticeable limitation of our review is the exclusion of recovered patients. It is a common practice for researchers to enroll recovered individuals in order to avoid the confounding effect of malnutrition and to detect permanent “scars” of the disease [160]. Our rationale behind this exclusion lies on the fact that recovery in ΑΝ is open to many clinical interpretations in the existing literature [161]. According to the DSM-V, full remission is achieved when none of the diagnostic criteria are fulfilled for a substantial period of time, without specifying the exact duration of being free of symptoms and without differentiating between adolescents and adults. For adolescents and youth in particular, recovery requires full weight restoration and normalization of eating pattern, pubertal progression and linear growth, if expected, as well as age-appropriate interpersonal, psychosocial, and occupational functioning [5]. Most studies that include recovered patients define recovery as weight restoration and maintenance for at least one year, thus providing comparability between their results. Nonetheless, given the fact that adolescents continue to grow and develop throughout puberty and into young adulthood, a “maintenance weight” restoration is far from characterizing a teen patient recovered [5]. A topic of significant questioning across AN neuroimaging is the potential confounding effect of co-morbid disorders, such as depression, stress disorder and OCD. This notion is further supported by the reported overlapping neuroimaging findings. As already mentioned, hippocampal atrophy is a common finding between AN patients and those with major depressive disorder. Similarly, depressive patients display changes in FA concerning mainly the genu and body of the corpus callosum and the corona radiata [84]. Likewise, adolescents with OCD are characterized by lower GM volume and CT of the parietal lobes [162]. Not only structural but also functional overlaps are apparent between AN and other psychiatric disorders. For instance, the hyperactivation of the PFC and the amygdala which are commonly reported in AN, are also features of the generalized stress disorder [163]. In addition, as mentioned before, altered functional connectivity between and within core RSNs characterizes many other psychiatric disorders, including OCD [162]. It is therefore quite difficult, if not impossible, to overcome the potential biases from co-morbid disorders, since these are the rule rather than the exception in AN patients.

The interpretation of findings needs to be considered in the light of several limitations characterizing all types of neuroimaging techniques. First of all, cohorts are consistently small and thus, with limited ability to control for potential confounding factors and to allow the generalization of the results. As highlighted by Thirion and colleagues, at least 20 subjects or more should be included in functional neuroimaging studies in order to have sufficient reliability [164], which can be quite difficult due to the high cost of the imaging procedures. Difficulty in enrolling patients usually results in heterogeneous samples in terms of demographic data and several clinical variables. Heterogeneity may exist even in samples including exclusively adolescents, due to the different neurodevelopmental stage of the participants. Even more profound is the sparsity of male patients. Interestingly, gender differences exist concerning cortical activation to taste in both the fasting state and satiety [139]. Thus, the neurobiological basis of the disease may differ considerably in males. Likewise, none of the included studies differentiated between the subtypes of the disease. However, it could be hypothesized that binge/purging behaviors may be related to different neurobiological paths from restrictive eating patterns. Another important factor that should be taken into consideration when studying adolescents pertains to the hormonal effects on the developing brain. For instance, research has shown that regional subcortical volumes are related to pubertal development, as measured by Tanner stage [165]. Additionally, a positive association has been reported between circulating estrogen levels and regional GM volumes [166]. Thus, given that pubertal development is partially dissociable from chronological age, matching study groups according to Tanner stage could be a reasonable approach [165]. Future systematic reviews could comparatively assess the present neuroimaging findings on AN versus other forms of malnutrition. Moreover, this systematic review subgrouped studies on the basis of imaging modalities; alternative subgrouping, as for instance according to methods of examining brain volumes, could have been performed but would not have allowed a clear link with the advantages and limitations of each modality.

Finally, as it has been already discussed, the interpretation of DTI findings is subject to additional limitations. First, differences in DTI parameters can emerge due to head motion during the scanning [167]. Only three studies have performed rigorous correction for head motion beyond the simple algorithm that is part of edgy current correction [56,59,60]. Second, dehydration could potentially affect diffusivity values, although the effects of dehydration on brain structure and function in eating disorders is an area of debate [168]. Studies used various methods to assess hydration status and some did not assess it at all (See Table 2). Urine specific gravity has been commonly used as a marker of hydration, however it may not be sufficient to diagnose hydration status and should be combined with other indices such as plasma and urine osmolarity [169]. Concluding, as discussed earlier, diffusion parameters could be affected by partial volume effect, at least in the fornix. Likewise, it could be hypothesized that WM tracts bordering the ventricular system, such as the corpus callosum and thalamic radiation could be affected as well. Consequently, the finding of reduced FA could be biased when ventricular volume has not been considered as a covariable.

Summarizing, our recommendations for future research are:

Since no standardized protocols are available, researchers are encouraged to follow proposed guidelines in order to increase validity, reliability and comparability of their results [168].

Enrolling adolescent patients at the earliest stage of the disease is the key to detect early biomarkers, before the confounding effects of malnutrition become apparent, albeit always considering developmental trajectories and puberty-related structural and functional deviations from normality.

Multi-center, longitudinal studies after long-term, physical and psychological recovery are proposed to conclusively disambiguate between trait-based variations and long-lasting effects of starvation. 

Likewise, studying populations at risk before the onset of the disease is essential to differentiate between premorbid trait markers and permanent scars of the disease.

Finally, multimodal neuroimaging techniques combining different methodological approaches for data analysis could offer a more comprehensive view of disease impact on brain. Following the same logic, researchers could ideally utilize both structural and functional imaging to address regions of interest.

## 5. Conclusions

This systematic review demonstrated potential associations between structural and functional alterations detected in young, anorectic patients and core features of the disease. Of course, the complexity of both the human brain and the disease does not allow the definite attribution of a symptom to a specific area dysfunction. Moreover, further research is needed in order to clarify whether these alterations are state-dependent or pre-morbid markers and therefore, potential targets for early detection and intervention.

## Figures and Tables

**Table 1:** Structural imaging studies—sample characteristics.

Study	Study Design	Subtypes	Males(%)	Mean Age (Years)	Age Range (Years)	Mean BMI (Kg/m^2^)	Duration of Follow-up (Months)	2nd Imaging Partici-Pation	2nd Mean BMI (Kg/m^2^)	Measure Adapted for 2nd Imaging	Criteria for Diagnosis	Duration of Illness (Months)	Patients under Medication (%)
**MRI**													
Katzman (1996)	Cross-sectional	AN-R: 13 HC: 8	0	15.2	13.3–17.0	15.6	-	-	-	-	DSM-III-R	11.3	0
Olivo (2018)	Cross-sectional	Atypical AN: 22 HC: 38	0	14.7	13–18	19.3	-	-	-	-	DSM-V	7.9	0
Myrvang (2018)	Cross-sectional	AN-R: 33 HC: 28	0	15.8	12.4–19.2	16.3	-	-	-	-	DSM-V	19.2	23.3
King (2015)	Cross-sectional	AN-R: 36 AN-BP: 4 HC: 40	0	15.9	12–23	14.8	-	-	-	-	DSM-IV	18	Not mentioned
Yue (2018)	Cross-sectional	AN-R: 17 AN-BP: 18 HC: 20	0	19.3	15–23	15.3	-	-	-	-	DSM-IV TR	33.3	0
Fujisawa (2015)	Cross-sectional	AN-R: 20 HC: 14	0	14.2	12–17	14.4	-	-	-	-	DSM-IV TR	23.55	0
Neumärker (2000)	Cross-sectional (longitudinal)	AN-R: 14 AN-BP: 4 HC: 25	0	14.5	13–16	14.9	Not mentioned	100%	17.8	Weight normalization	ICD-10	267.8	Not mentioned
Castro-Fornieles (2009)	Cross-sectional (longitudinal)	AN-R: 9 AN-BP: 3 HC: 9	8.3	14.5	11–17	14.8	6	100%	18.8	Weight normalization.	DSM-IV TR	8.3	8.3
Monzon (2017)	Cross-sectional (longitudinal)	AN: 26 HC: 10	0	16.5	14 -19	16.7	2	38%	18.9	Weight gain	DSM-V	Less than 36	Not mentioned
Golden (1996)	Cross-sectional (longitudinal)	AN: 12 HC: 12	0	16.1	11–22	14.3	11	100%	17.9	Weight normalization	DSM-III	Not mentioned	Not mentioned
Akgül (2016)	Cross-sectional (longitudinal)	AN: 9 HC: 9	11	15.8	13–21	16.3	14	100%	19.2	Weight normalization	DSM-IV	8.7	0
Bernardoni (2016)	Cross-sectional (longitudinal)	AN-R: 43 AN-BP: 4 HC: 35	0	15.5	12–23	14.8	3	35	18.7	Weight gain	DSM-IV	Not mentioned	0
Katzman (1997)	Cross-sectional (longitudinal)	AN: 6 HC: 6	0	17.0	15–19	15.9	24	100%	23.0	Weight normalization	DSM-III-R	22.5	0
**MRS**													
Schlemmer (1997)	Cross-sectional	AN-R: 8 AN-BP: 2 HC: 17	0	16.0	14.9–19	14.7	-	-	-	-	DSM-IV	Not mentioned	0
Blasel (2012)	Cross-sectional	AN-R: 19 AN-BP: 2 HC: 29	0	14.4	11–17	14.4	-	-	-	-	DSM-IV	11	0
Castro-Fornieles (2007)	Cross-sectional (longitudinal)	AN-R: 9 AN-BP: 3 HC: 12	8.3	14.5	11–17	14.8	7	100%	-	Weight normalization	DSM-IV-TR	Not mentioned	8.3
**MRI-DTI**													
Pfuhl (2016)	Cross-sectional	AN: 35 HC:62	0	16.1	12–24	14.7	-	-	-	-	DSM-IV	Not mentioned	0
Hu (2017)	Cross-sectional	AN-R: 8 HC:14	0	17.6	15–22	14.3	-	-	-	-	DSM-IV	10.5	Not mentioned
Gaudio (2017)	Cross-sectional	AN-R: 14 HC:15	0	15.7	13–18	16.2	-	-	-	-	DSM-IV-TR	4.9	0
K. E.Travis (2015)	Cross-sectional	AN-R: 15 HC:15	0	16.6	14–18	16	-	-	-	-	DSM-IV	16.3	2
K. Vogel (2016)	Cross-sectional (longitudinal)	AN-R: 19 AN-BP: 3 HC: 21	0	15.03	10–18	15.36	4.76	41%	17.4	Weight gain	DSM-IV	13.49	1
G. Olivo (2018)	Cross-sectional	AAN: 25 HC:25	0	14.08	13–18	18.6	-	-	-	-	DSM-V	8.4	0
Von Schwanenflug (2018)	Cross-sectional (longitudinal)	AN-R: 53 AN-BP: 3 HC:60	0	15.8	12–27	14.7	3	83%	18.7	Weight gain	DSM-IV	14.5	Not mentioned

**Table 2:** Structural imaging studies—methods, main findings and clinical interpretations.

Study	Method and Procedure	Data Analysis	Hydration before Imaging for DTI Studies	Presentation of the Main Findings	Clinical Interpretations
MRI					
Katzman (1996)	MRI 1.5T Tested at one time point.	Not mentioned		Significantly larger total CSF volume and reduced total GM and WM volumes. Alterations correlated with BMI and cortisol levels. No correlation with disease duration.	No clinical interpretations. Deficits in GM volume were associated with severity but not disease duration and were related to hypercortisolemia.
Olivo (2018)	MRI 3T Tested at one time point.	Voxel based morphometry (VBM)		Total GM, WM, and CSF volumes were not significantly different between groups.	Τhe preservation of GM volume might indeed differentiate atypical AN from AN. Alternatively, there may be a weight cut-off under which GM alterations become obvious.
Myrvang (2018)	MRI 3T Tested at one time point.	Magnetization Prepared—RApid Gradient Echo (MPRAGE)-sequence		Statistically significant volume reduction in GM, total hippocampal volume and in all hippocampal subfields apart from fissure.	Hippocampal atrophy may be attributed to hypercortisolemia due to high levels of stress.
King (2015)	MRI 3T Tested at one time point.	Source based morphometry (SBM)		Significant GM thickness reduction in a total of 86% of the cortical surface, apart from bilateral temporal pole and entorhinal cortex. Reduced volume of nucleus accumbens, amygdala, cerebellum, hippocampus, putamen and thalamus.	A correlation was found between cortical thickness and “drive for thinness” in a broad region of the right lateral occipitotemporal cortex. The normal neurodevelopmental trajectory of cortical thickness (CT) across adolescence and young adulthood may be interrupted in AN.
Yue (2018)	MRI 3T Tested at one time point.	Not mentioned		Significantly reduced total GM volume and ventricular enlargement. Reduced thalamus volume CT in the left precuneus and a larger ratio of caudate volume.	The relative preservation of caudate volume and reduced CT of the left precuneus may be involved in body image distortion.
Fujisawa (2015)	MRI 3T Tested at one time point.	VBM		Significant volume decreases in total GM as well as in bilateral inferior frontal gyrus (IFG) (19,1% left and 17,6% right). Significant correlations were found between regional reduction of GM in the bilateral IFG and age, BMI and age at disease onset.	Volumetric decreases in the IFG might explain the impulsive behaviors observed in patients with AN.
Neumärker (2000)	MRI 1.5T Testedat three time points: at admission (T1), with 50% weight restoration (T2), with normal weight (T3).	Not mentioned		**T1:** Significant larger lateral ventricles and wider fissures of Sylvius bilaterally. Mesencephalon was also markedly reduced. **T2&T3:** Reduced mesencephalon size persisted.	Volumetric alterations were related to the degree of impairment in arithmetic performance. Intact number processing abilities may be a good predictor for weight restoration.
Castro-Fornieles (2009)	MRI 1.5T Tested at two time points: before treatment (T1) and after weight recovery (T2).	VBM		**T1:** Lower global GM and higher CSF volumes and not statistically significant differences in WM. In regional VBM study, significantly decreased GM was observed in bilateral parietal, right temporal cortex and cingulum. **T2:** Decreased GM volume remained in cingulum, not to the same extent as in the first assessment.	Overall, GM reduction at first assessment correlated with Rey Complex Figure Test copy time, indicating a relationship to slowness in complex mental processing.
Monzon (2017)	MRI 3T 26 AN(T1) patients evaluated at the beginning, 10 AN(T2) patients re-examined after reaching at least 85% of expected body weight.	VBM		**T1:** Significantly reduced GM volume in OFC, dlPFC, mPFC, insular cortex and hippocampus, anterior cingulate cortex (ACC), medial cingulate cortex (MCC), posterior cingulate cortex (PCC) and the precuneus bilaterally. Additionally, in bilateral amygdala and thalamus. No significant difference in total brain volume between groups. **T2:** Significantly reduced GM volume remained in ACC, caudate nucleus and right hippocampus. GM volume increase after weight gain in thalamus was negatively correlated to the presence of eating concern symptoms, while in left OFC was negatively correlated to shape-concern symptoms evaluated by the EDE-Q.	Alterations found in PFC, insular and cingulate cortices, hippocampal region, amygdala and parietal cortex could explain distorted body image, emotional disturbances and cognitive deficits.
Golden (1996)	MRI 1T Tested at two time points: before treatment (T1) and after weight gain (T2).	Not mentioned		**T1:** Ventricular enlargement, especially of the third ventricle. **T2:** Significantly decreased total ventricular volume. An inverse relationship was found between ventricular volume and BMI.	Atrophy of the cerebral cortex may occur as a result of decreased protein synthesis caused by malnutrition. Structural changes and cognitive functioning seem to improve with weight gain.
Akgül (2016)	MRI 1.5T-MTI Tested at two time points: T1 at admission, T2 after weight recovery.	Regions of interest (ROIs)		**T1:** Magnetization Transfer Ratio (MTR) did not differ. MRI identified widening of the cerebral sulci in 7 patients with no other gross abnormalities. (ROIs: Left dlPFC, left cerebellar hemisphere, thalamus, amygdala, pons, corona radiata). **T2:** MTR did not differ.	No clinical interpretations. Adiposity-related variations in phospholipid composition of brain lipids during adolescence could be related to the reversibility of functional impairment.
Bernardoni (2016)	MRI 3T Tested at two time points: T1 at admission, T2 after weight recovery.	SBM-ROIs		**T1:** Global cortical thinning. **AN(T1) vs. AN(T2):** 84% of CT restored. **AN(T2) vs. HC:** CT normalised apart from left temporal pole and enthorhinal cortex. Subcortical GM volume was increased in all ROIs apart from pallidum where a decrease was observed.	Normalization of CT following partial weight restoration is independent of improvements in psychopathology.
Katzman (1997)	MRI 1.5T Tested at two time points: at low weight (T1) and at normal weight (T2) 2–3 years later.	Not mentioned		**T1:** GM and WM volume decrease and ventricular enlargement. **T2:** Findings persisted apart from WM volume decrease. Increase of GM volume correlated with BMI increase.	Hypercortisolemia may lead to neuronal damage and persistent brain abnormalities.
MRS					
Blasel (2012)	MRI 3T and MRS Tested at one time point.	Separate analysis of region1: anterior region rostral of the anterior commissure & region 2: posterior region dorsal of the anterior commissure.		No difference between GM fraction. WM fraction was significantly lower to region 2. Significant differences in metabolite concentrations were determined in GM with higher concentrations of tCho, tCr, tNAA, Glx. No difference was found in WM metabolites. MI concentrations did not differ between patients and controls.	The Glx increase may indicate a psychiatric or neurodegenerative origin of AN rather than the result of nutrition depletion.
Castro-Fornieles (2007)	MRI 1.5T and MRS Tested at two time points: T1 before treatment and T2 after weight recovery.	Not mentioned		**T1:** Significantly lower NAA, Glx and mI. No difference was found in the concentration of Cr and Chol. A positive correlation was reported between NAA & T3 and NAA & Wechsler Intelligence Scale for children (WISC). No difference in metabolites concentration between males and females. **T2:** A statistically significant increase in NAA and a non-significant increase in Glx in frontal GM.	No clinical interpretations.
Schlemmer (1997)	MRI 1.5T and MRS Tested at one time point.	Two ROIs: the parieto-occipital WM and the thalamus.		A 25% elevation of Cho/Cr and a 25% depression of NAA/Cho were observed in the parieto-occipital WM. No statistically significant differences were found in thalamus. No correlations were found between the metabolic ratios and age, weight or BMI.	No clinical interpretations. The abnormal phospholipid metabolism of membranes might be responsible for brain atrophy.
MRI-DTI					
Pfuhl (2016)	DTI, MRI Tested at one time point.	Global tractography	Urine specific gravity	No significant volumetric differences or microstructural abnormalities in 18 WM tracts. All four diffusivity indices were evaluated (FA, MD, AD, RD).	The preserved WM microstructure may explain why adolescents often do not show marked impairment in executive functioning.
Hu (2017)	DTI Tested at one time point.	VBM	At least 1 week of supervised meals and hydration.	Decreased FA in the left superior frontal gyrus, medial frontal gyrus, ACC, middle frontal gyrus, IFG, thalamus and bilateral insula. Positive correlations between the FA of the left IFG, insula, thalamus and BMI.	WM alterations in prefrontal cortex, parietal lobe and subcortical regions may be associated with impaired cognitive functions.
Gaudio (2017)	DTI Tested at one time point.	VBM	Not assessed	Decreased FA in the left anterior and superior corona radiata and in the SLF. Decreased AD in the left superior and anterior corona radiata and in the SLF bilaterally, external capsule, posterior limb of the internal capsule and posterior thalamic radiation. No differences in MD, RD. No significant correlations.	WM alterations may be involved in impaired cognitive flexibility and body image distortion.
Travis (2015)	DTI Tested at one time point.	Tractography, relaxometry	Not assessed	Twenty-six WM tracts were identified, 9 bilateral cerebral and 8 subdivisions of the corpus callosum. FA was found decreased in 4 of 26 tracts (including bilateral fimbria—fornix and right SLF and motor subdivisions of corpus callosum) and increased in 2 (including right anterior thalamic radiation and left SLF). R1 was decreased in 11 of 26 tracts mainly in corticospinal tracts and subdivisions of the corpus callosum—body and splenium. No significant associations between BMI and clinical measures.	WM alterations seem to be related to myelin quality, affecting cognitive, emotional and social functions.
Vogel (2016)	DTI Tested at one time point.	TBSS	Urine specific gravity	**T1:** Increased FA in bilateral frontal, parietal and temporal areas, including bilateral superior corona radiata, corpus callosum, anterior and posterior thalamic radiation, anterior and posterior limb of internal capsule and left inferior longitudinal fasciculus. FA increase due to reduced RD, not altered AD. Most areas with FA increase exhibited reduced MD. **T2:** No differences in FA after weight rehabilitation. Higher FA was associated with faster weight loss.	The different pattern of WM microstructural changes in adolescents compared to adults may reflect a different susceptibility and reaction to semi starvation in the still developing brain or a time-dependent pathomechanism differing with extent of chronicity.
Olivo (2018)	DTI Tested at one time point.	TBSS	Patients were instructed to eat before the scanning.	No differences detected in diffusivity indices (FA, MD, RD, AD).	Preserved WM microstructure in patients with atypical AN suggests that alterations observed in full syndrome may constitute state-related consequences of severe weight loss.
Von Schwanenflug (2019)	DTI Tested at two time points: At baseline (T1) and after partial weight restoration (T2).	TBSS	Urine specific gravity	**T1:** In acAN significantly decreased FA and increased MD, AD, RD in corpus callosum, mainly in the body and increased FA in the right corticospinal tract. Additionally, increased FA in the right SLF. **T2:** After partial weight restoration significantly increased FA and dicreased MD, AD, RD in the fornix extending into bilateral optic radiation. No clinical correlations.	The decreased FA in corpus callosum may contribute to the distorted body image.

**Table 3:** Functional imaging studies—sample characteristics.

Study	Study Design	Sybtype	Males(%)	Mean Age (Years)	Age Range (Years)	Mean BMI (Kg/m^2^)	Duration of Follow-up (Months)	2nd Imaging Participation	2nd Mean BMI (Kg/m^2^)	Measure Adapted for 2nd Imaging	Criteria for Diagnosis	Duration of Illness (Months)	Patients under Medication (%)
SPECT													
Kojima (2005)	Cross-sectional (longitudinal)	AN-R: 12 HC: 11	0	18.6	15.1–22.1	12.5	3.46	100%	15.6	Weight gain	DSM-IV	Not mentioned	Not mentioned
Takano (2001)	Cross-sectional	AN-R: 8 AN-BP: 6 HC: 8	0	21.2	-	14.0	-	-	-	-	DSM-IV	16.8	0
Matsumoto (2006)	Longitudinal	AN-R: 5 AN-BP: 3 HC: 8	0	18.5	12.3–24	12.9	6	100%	18.8	Weight normalization.	DSM-IV	28	0
Komatsu (2010)	Longitudinal	AN: 10 HC: 10	0	13.2	11.0–14.3	13.1	3	100%	16.6	Weight gain	DSM-IV	Early onset	0
fMRI									-				
S. Gaudio (2015)	Cross-sectional	AN-R: 16 HC:16	0	15.8	13–18	16.2	-	-	-	-	DSM-IV TR	4	0
I. Boehm (2014)	Cross-sectional	AN-R: 33 AN-BP: 2 HC: 35	0	16.1	12–23	14.8	-	-		-	DSM-IV	18.9	0
F. Amianto (2013)	Cross-sectional	AN-R: 12 HC:10	0	20.0	16–24	16.3	-	-	-	-	DSM-IV	11.5	0
S. Gaudio (2018)	Cross-sectional	AN-R: 15 HC:15	0	15.7	13–18	16.1	-	-		-	DSM-IV TR	4	0
D. Geisler (2015)	Cross-sectional	AN: 35 HC:35	0	16.1	12–23	14.8	-	-	-	-	DSM-IV	18.9	0
S. Ehrlich (2015)	Cross-sectional	AN-R: 33 AN-BP: 2 HC: 35	0	16.1	12–23	14.8	-	-	-	-	DSM-IV	18.9	0

**Table 4:** Functional imaging studies—methods, main findings and clinical interpretations.

Study	Method and Procedure	Data Analysis	Presentation of the Main Findings	Clinical Interpretations
SPECT				
Kojima (2005)	SPECT Tested at two time points: at baseline (T1) and after weight recovery (T2).	(HMPAO)	**AN (T1) vs. HC:** Decreased rCBF in AN in the bilateral frontal lobes, including the ACC, PCC, bilateral precentral gyri, right insula, and right lingual gyrus. A positive correlation between the rCBF and BMI in the occipital lobe was found. **AN (T2) vs. HC:** Significant increases in the right parietal lobe and the left superior frontal gyrus. Decreases in the left superior temporal gyrus, left putamen, right IFG, right amygdala, and right cerebellum.	Hypoperfusion in the ACC and the parietal lobe may be associated not only with low body weight but also with abnormal brain functions relative to clinical features of AN.
Takano (2001)	SPECT Tested at one time point.	I-123-MIBG SPM approach	**AN vs. HC:** Hypoperfusion in the mPFC and ACC. Hyperperfusion in the thalamus and amygdala-hippocampus complex.	Hypoperfusion of the ACC may reflect depressive symptoms, while hyperactivity of the thalamus may be associated with chronic and refractory AN.
Matsumoto(2006)	SPECT Tested at two time points: at baseline (T1) and before discharge (T2).	123I-IMP	**AN-T1 vs. AN-T2:** Significant increase in rCBF in right dlPFC and medial parietal cortex including the precuneus and in the PCC. At the same lower threshold (*p* < 0.002) rCBF in the ACC and mPFC increased to an almost significant level. Significant decrease of rCBF in the right putamen.	Changes in rCBF may be associated with the improvement of interoceptive awareness following treatment.
Komatsu (2010)	SPECT Tested at two time points: at baseline (T1) and after 3 months (T2).	123I-IMP SPM approach	**AN-T1 vs. AN-T2:** Significant increased rCBF in bilateral parietal lobes and right PCC. No regions of decreased rCBF. A positive correlation between BMI and rCBF in right thalamus, right parietal lobe and right cerebellum.	PCC activation after weight gain might reflect affective changes for eating motivation during the recovery process of early-onset AN.
fMRI				
Gaudio (2015)	fMRI + MRI Tested at one time point.	whole brain ICA analysis	**AN vs. HC:** Eight networks were identified. Statistically significant reduced connectivity between the Executive control network (ECN) and the ACC. The decrease in functional connectivity in the ACC was positively correlated with BMI and negatively correlated with drive for thinness, perfectionism and harm avoidance scores. No significant differences in GM volumes.	The decreased functional connectivity between the ECN and the ACC could explain the cognitive inflexibility in relation to body image.
Boehm (2014)	fMRI Tested at one time point.	ICA network based analysis	**AN vs. HC:** The networks of interest were the Fronto parietal network (FPN), DMN, Salience network (SN), visual and sensory-motor network. Increased functional connectivity between the angular gyrus and the FPN and between the anterior insula and the DMN. Positive correlations for both networks (DMN, FPN) with self-report measures in healthy controls. Functional connectivity in the anterior insula was positively associated with interoceptive difficulties in HC.	Increased functional connectivity within the FPN might be related to excessive cognitive control. The increased functional connectivity of insula with the DMN may mirror difficulties to disengage from thoughts about food and body appearance when not engaged in a task.
Amianto (2013)	fMRI, MRI Tested at one time point.	ICA network based analysis	**AN vs. HC:** Within the cerebellar intrinsic connectivity network, a greater connectivity was found with insulae, temporal poles, vermis and paravermis and a lesser connectivity with parietal lobe. Additionally, GM volume reduction in cerebellar hemispheres, cingulate cortex, precuneus and OFC.	The vermian hyper-connection could be linked to some psychopathological core features, such as “drive for thinness” which express the dissatisfaction with body weight. The cerebellar-parietal network dysfunction could be related to the disturbances in the body image perception. A stronger connection between cerebellum and temporal lobes may be related to greater emotional activation elicited by social behaviors in subjects with AN.
Gaudio (2018)	fMRI, MRI Tested at one time point.	Graph analysis whole brain and network based	**AN vs. HC:** Decreased connectivity in the sub-network including the left and right rostral ACC, left paracentral lobule, left cerebellum, left posterior insula, left medial orbito-frontal gyrus and right superior occipital gyrus. No significant differences in GM, WM, and CSF volumes.	The altered sub-network functional connectivity may sustain an altered self-body imge through an impaired integration of somatosensory, visual and interoceptive signals.
Geisler (2015)	fMRI Tested at one time point.	Graph analysis whole brain	**AN vs. HC:** Decreased functional connectivity in the thalamo-insular subnetwork. Longer average routes between nodes and more nodes with a similar connectedness link together. Additionally, altered global network architecture.	The altered network global topology indicates wide-scale disturbance in information flow across brain networks. The local thalamo-insular network disruption may explain the impaired integration of visuospatial and homeostatic signals.
Ehrlich (2015)	fMRI Tested at one time point.	Network based statistics	**AN vs. HC:** Reduced functional connectivity in the thalamo-insular network (in particular in a subnetwork consisting of the thalamus, amygdala, basal ganglia, fusiform gyrus and posterior insula).	The decreased functional connectivity in the thalamo-insular network may explain the striking discrepancy between patient’s actual and perceived internal body state.

## Data Availability

Data is contained within the article.

## Supplementary Materials

The following are available online at https://www.mdpi.com/2227-9067/8/2/137/s1, Table S1: Studies excluded, with their reason for exclusion. Table S2a: Evaluation of the eligible studies with Newcastle-Ottawa scale- Case-Control studies. Table S2b: Evalusation of the eligible studies with Newcastle-Ottawa scale- Cohort studies.

## Abbreviations

ACC: Anterior Cingulate cortex; AD: Axial Diffusivity; AN: Anorexia Nervosa; AN-BP: Anorexia nervosa-Binge Purge type; AN-R: Anorexia Nervosa-Restricting type; BMI: Body Mass Index; Cho: Choline; CSF: Cerebrospinal Fluid; Cr: Creatine; CT: Cortical Thickness; dlPFC: Dorsolateral Prefrontal Cortex; DMN: Default Mode Network; DSM: Diagnostic and Statistical Manual of Mental Disorders; Dstr: Dorsal striatum; DTI: Diffusion Tensor Imaging; ECN: Executive Control Network; FA: Fractional Anisotropy; fMRI: Functional Magnetic Resonance Imaging; FPN: Fronto-Parietal Network; Glutamate/Glutamine: Glx; GM: Grey Matter; HC: Healthy Control participants; ICA: Independent component analysis; IFG: Inferior Frontal Gyrus; MCC: Medial Cingulate Cortex; MD: Mean Diffusivity; mI: myo-Inositol; MFG: Middle Frontal Gyrus; mPFC: Medial Prefrontal Cortex; MRI: Magnetic Resonance Imaging; MRS: Magnetic Resonance Spectroscopy; MTR: Magnetization Transfer Ratio; NAA: N-acetyl-aspartate; OCD: Obsessive Compulsive Disorder; OFC: Orbitofrontal Cortex; PCC: Posterior Cingulate Cortex; PFC: Prefrontal Cortex; PRISMA: Preferred Reporting Items for Systematic Reviews and Meta-Analyses; PTSD: Posttraumatic Stress Disorder; PVE: Partial Volume Effect; RD: Radial Diffusivity; rCBF: Regional Cerebral Blood Flow; ROIs: Regions Of Interest; RSFC: Resting State Functional Connectivity; RSN: Resting State Network; SBM: Surface Based Morphometry; SLF: Superior Longitudinal Fasciculus; SMA: Supplementary Motor Area; SN: Salience Network; SPECT: Single- Photon Emission Computed Tomography; SPM:s Statistical Parametric Mapping; TBSS:tract-based spatial statistics; tCho: total Choline; tCr: total Creatine; VBM: Voxel Based Morphometry; WISC: Wechsler Intelligence Scale for Children; WM: White Matter.
