# Supplementary material for: Neuroimaging Findings in Adolescents and Young Adults with Anorexia Nervosa: A Systematic Review
Source: Children (Basel). 2021 Feb 12;8(2):137. doi: 10.3390/children8020137 (PMC7918703; doi:10.3390/children8020137)
Supplement: Supplementary file 1 [file children-08-00137-s001.pdf]

Table S1. Studies excluded, with their reason for exclusion

Study	*Title*	*Reason for exclusion*
Bomba (2013)	Impact of speed and magnitude of weight loss on the development of brain trophic changes in adolescents with anorexia nervosa: a case control study.	Comorbidity (patient with celiac disease)
Swayze (1996)	Reversibility of brain tissue loss in anorexia nervosa assessed with a computerized Talairach 3-D proportional grid	Diagnosis of AN is not established through official criteria.
Boto (2017)	Evaluating anorexia-related brain atrophy using MP2RAGE-based morphometry.	Diagnosis of AN is not established through official criteria.
Dolan (1988)	Structural brain changes in patients with anorexia nervosa	Diagnosis of AN is not established through official criteria.
Artmann (1985)	Reversible and non-reversible enlargement of cerebrospinal fluid spaces in anorexia nervosa	Diagnosis of AN is not established through official criteria.
Kohlmeyer (1983)	Computed tomography of anorexia nervosa.	Diagnosis of AN is not established through official criteria.
Wallace (2019)	Subclinical eating disorder traits are correlated with cortical thickness in regions associated with food reward and perception.	Diagnosis of AN is not established through official criteria.
Cowdhury (2003)	Early-Onset Anorexia Nervosa: Is There Evidence of Limbic System Imbalance?	Diagnosis of AN is not established through official criteria.
Rothemud (2011)	Compulsivity Predicts Fronto Striatal Activation in Severely Anorectic Individuals	fMRI after stimulation.
Schulte-Rüther (2012)	Theory of Mind and the Brain in Anorexia Nervosa: Relation to Treatment Outcome	fMRI after stimulation.
Lao-Kaim (2014)	Functional MRI Investigation of Verbal Working Memory in Adults With Anorexia Nervosa	fMRI after stimulation.
Bischoff-Grethe (2013)	Altered Brain Response to Reward and Punishment in Adolescents With Anorexia Nervosa	fMRI after stimulation.
Fladung (2013)	Role of the Ventral Striatum in Developing Anorexia Nervosa	fMRI after stimulation.
McFadden (2014)	Reduced Salience and Default Mode Network Activity in Women With Anorexia Nervosa	fMRI after stimulation.
Nagahara (2014)	A Tract-Based Spatial Statistics Study in Anorexia Nervosa: Abnormality in the Fornix and the Cerebellum	fMRI after stimulation.
Lao-Kaim (2015)	Aberrant Function of Learning and Cognitive Control Networks Underlie Inefficient Cognitive Flexibility in Anorexia Nervosa: A Cross-Sectional fMRI Study	fMRI after stimulation.
Frank (2016)	Altered structural and effective connectivity in anorexia and bulimia nervosa in circuits that regulate energy and reward homeostasis.	fMRI after stimulation.
Hildebrandt (2018)	Evidence of prefrontal hyperactivation to food-cue reversal learning in adolescents with anorexia nervosa.	fMRI after stimulation.
Bohon (2019)	Performance and brain activity during the Wisconsin Card Sorting Test in adolescents with obsessive-compulsive disorder and adolescents with weight-restored anorexia nervosa.	fMRI after stimulation.
Castro-Fornieles (2019)	Functional MRI with a set-shifting task in adolescent anorexia nervosa: A cross-sectional and follow-up study.	fMRI after stimulation.
Horster (2020)	A Neglected Topic in Neuroscience: Replicability of fMRI Results With Specific Reference to ANOREXIA NERVOSA	fMRI after stimulation.
Doose (2020)	Strengthened default mode network activation during delay discounting in adolescents with anorexia nervosa after partial weight restoration: A longitudinal FMRI study	fMRI after stimulation.
Castro-Fornieles (2010)	A cross-sectional and follow-up functional MRI study with a working memory task in adolescent anorexia nervosa	fMRI after stimulation.
Leppanen (2017)	Blunted neural response to implicit negative facial affect in anorexia nervosa	fMRI after stimulation.
Seidel (2017)	Processing and regulation of negative emotions in anorexia nervosa: An fMRI study	fMRI after stimulation.
Zutphen (2019)	Intimate stimuli result in fronto-parietal activation changes in anorexia nervosa	fMRI after stimulation.

Maier (2019)	Neurobiological signature of intimacy in anorexia nervosa	fMRI after stimulation.
Steward (2020)	Dorsolateral prefrontal cortex and amygdala function during cognitive reappraisal predicts weight restoration and emotion regulation impairment in anorexia nervosa	fMRI after stimulation.
Horndasch(2020)	Neural mechanisms of perceptive and affective processing of body stimuli in anorexia nervosa - are there developmental effects?	fMRI after stimulation.
Olivo(2019)	Functional connectivity underlying hedonic response to food in female adolescents with atypical AN: the role of somatosensory and salience networks.	fMRI after stimulation.
Sheng (2015)	Cerebral Perfusion Differences in Women Currently With and Recovered From Anorexia Nervosa	Patients under psychotropic medication.
Kingston (1996)	Neuropsychological and structural brain changes in anorexia nervosa before and after refeeding	Patients under psychotropic medication.
Yonezawa (2008)	No differences are seen in the regional cerebral blood flow in the restricting type of anorexia nervosa compared with the binge eating/purging type	Patients under psychotropic medication.
Olivo (2018)	Reduced resting-state connectivity in areas involved in processing of face-related social cues in female adolescents with atypical anorexia nervosa.	Patients under psychotropic medication.
Zucker (2017)	The Clinical Significance of Posterior Insular Volume in Adolescent Anorexia Nervosa.	Patients under psychotropic medication.
Gaudio (2011)	Gray matter decrease distribution in the early stages of Anorexia Nervosa restrictive type in adolescents.	Patients under psychotropic medication.
Kazlouski (2011)	Altered fimbria-fornix white matter integrity in anorexia nervosa predicts harm avoidan	Patients under psychotropic medication.
Mainz (2012)	Structural Brain Abnormalities in Adolescent Anorexia Nervosa Before and After Weight Recovery and Associated Hormonal Changes	Patients under psychotropic medication.
Frank (2013)	Localized Brain Volume and White Matter Integrity Alterations in Adolescent Anorexia Nervosa	Patients under psychotropic medication.
Seitz (2015)	Brain Volume Reduction Predicts Weight Development in Adolescent Patients With Anorexia Nervosa	Patients under psychotropic medication.
Phillipou (2016)	Resting State Functional Connectivity in Anorexia Nervosa	Patients under psychotropic medication.
Phillipou (2018)	Differences in regional grey matter volumes in currently ill patients with anorexia nervosa.	Patients under psychotropic medication.
Lavagnino (2018)	Cortical thickness patterns as state biomarker of anorexia nervosa.	Patients under psychotropic medication.
Kaufmann (2017)	Fornix Under Water? Ventricular Enlargement Biases Forniceal Diffusion Magnetic Resonance Imaging Indices in Anorexia Nervosa.	Patients under psychotropic medication.
Miles (2018)	Subcortical volume and cortical surface architecture in women with acute and remitted anorexia nervosa: An exploratory neuroimaging study.	Patients under psychotropic medication.
Rastam (2001)	Regional cerebral blood flow in weight-restored anorexia nervosa: a preliminary study.	Psychiatric co-morbidity.
Lankenau (1985)	Cranial CT Scans in Eating Disorder Patients and Controls	Psychiatric co-morbidity.
Phillipou (2018)	White matter microstructure in anorexia nervosa.	Psychiatric co-morbidity.
Ehrlich (2015)	Reduced Functional Connectivity in the Thalamo-Insular Subnetwork in Patients With Acute Anorexia Nervosa	Psychiatric co-morbidity.
Biezonski (2016)	Evidence for Thalamocortical Circuit Abnormalities and Associated Cognitive Dysfunctions in Underweight Individuals With Anorexia Nervosa	Psychiatric co-morbidity.
Krieg (1989)	Brain Morphology and Regional Cerebral Blood Flow in Anorexia Nervosa	Sample out of age range.
Uniacke (2019)	Resting-state connectivity within and across neural circuits in anorexia nervosa	Sample out of age range.
Cha (2016)	Abnormal Reward Circuitry in Anorexia Nervosa: A Longitudinal, Multimodal MRI Study	Sample out of age range.
Burkert (2019)	Body image disturbances, fear and associations with the amygdala in anorexia nervosa.	Sample out of age range.

Bernardoni (2018)	Nutritional status affects cortical folding: Lessons learned from anorexia nervosa	Sample out of age range.
Doraiswamy (1990)	A brain magnetic resonance imaging study of pituitary gland morphology in anorexia nervosa and bulimia.	Sample out of age range.
Boghi (2011)	In vivo evidence of global and focal brain alterations in anorexia nervosa.	Sample out of age range.
Brooks (2011)	Restraint of appetite and reduced regional brain volumes in anorexia nervosa: a voxel-based morphometric study.	Sample out of age range.
Burkert (2015)	Structural hippocampal alterations, perceived stress, and coping deficiencies in patients with anorexia nervosa.	Sample out of age range.
Agata (2015)	Brain correlates of alexithymia in eating disorders: A voxel-based morphometry study.	Sample out of age range.
Amianto (2013)	Brain volumetric abnormalities in patients with anorexia and bulimia nervosa: a voxel-based morphometry study.	Sample out of age range.
Lavagnino (2016)	The relationship between cortical thickness and body mass index differs between women with anorexia nervosa and healthy controls.	Sample out of age range.
Khalsa (2016)	Mammillary body volume abnormalities in anorexia nervosa.	Sample out of age range.
Hoffman (1989)	Cerebral atrophy in anorexia nervosa: a pilot study.	Sample out of age range.
Audenaert (2003)	Decreased 5-HT2a Receptor Binding in Patients with Anorexia Nervosa	Sample out of age range.
Joos (2010)	Voxel-based morphometry in eating disorders: Correlation of psychopathology with grey matter volume	Sample out of age range.
Friederich (2012)	Grey matter abnormalities within cortico-limbic-striatal circuits in acute and weight-restored anorexia nervosa patients	Sample out of age range.
Nickel (2018)	Recovery of cortical volume and thickness after remission from acute anorexia nervosa	Sample out of age range.
Frampton (2010)	Do Abnormalities in Regional Cerebral Blood Flow in Anorexia Nervosa Resolve after Weight Restoration?	Sample out of age range.
Santos (2018)	Registration-based methods applied to serial high-resolution T1-weighted T magnetic resonance imaging for the assessment of brain volume changes in anorexia nervosa of the restricting type	Sample out of age range.
Mustafa (1992)	Subcorticai Brain Anatomy in Anorexia and Bulimia	Sample out of age range.
Palazidou (1999)	Neuroradiological and neuropsychological assessment in anorexia nervosa	Sample out of age range.
Nauro (2001)	Decreases in blood perfusionof the anterior cingulate gyri in Anorexia Nervosa Restricters assessed by SPECT image analysis	Sample out of age range.
Swayze (2002)	rain Tissue Volume Segmentation in Patients with Anorexia Nervosa before and after Weight Normalization	Sample out of age range.
Connan (2006)	Hippocampal volume and cognitive function in anorexia nervosa	Sample out of age range.
McCormick (2008)	Implications of Starvation-Induced Change in Right Dorsal Anterior Cingulate Volume in Anorexia Nervosa	Sample out of age range.
Suchan (2010)	Reduction of gray matter density in the extrastriate body area in women with anorexia nervosa	Sample out of age range.
Frieling (2012)	Microstructural Abnormalities of the Posterior Thalamic Radiation and the Mediodorsal Thalamic Nuclei in Females With Anorexia Nervosa--A Voxel Based Diffusion Tensor Imaging (DTI) Study	Sample out of age range.
Favaro (2012)	Disruption of Visuospatial and Somatosensory Functional Connectivity in Anorexia Nervosa	Sample out of age range.
Via (2014)	Disruption of Brain White Matter Microstructure in Women With Anorexia Nervosa	Sample out of age range.
Kullmann (2014)	Aberrant Network Integrity of the Inferior Frontal Cortex in Women With Anorexia Nervosa	Sample out of age range.
Favaro (2014)	Effects of Obstetric Complications on Volume and Functional Connectivity of Striatum in Anorexia Nervosa Patients	Sample out of age range.
Favaro (2015)	Gyrification Brain Abnormalities as Predictors of Outcome in Anorexia Nervosa	Sample out of age range.
Canna (2016)	Interhemispheric functional connectivity in anorexia and bulimia nervosa.	Sample out of age range.

Scaife (2017)	Reduced Resting-State Functional Connectivity in Current and Recovered Restrictive Anorexia Nervosa.	Sample out of age range.
Kohmura (2017)	Regional decrease in gray matter volume is related to body dissatisfaction in anorexia nervosa.	Sample out of age range.
Boto(2019)	Cerebral Gray and White Matter Involvement in Anorexia Nervosa Evaluated by T1, T2, and T2* Mapping.	Sample out of age range.
Miles (2019)	White matter microstructure in women with acute and remitted anorexia nervosa: an exploratory neuroimaging study.	Sample out of age range.
Fonville (2013)	Alterations in brain structure in adults with anorexia nervosa and the impact of illness duration.	Sample out of age range.
Maier (2020)	Insular cell integrity markers linked to weight concern in anorexia nervosa—an MR-spectroscopy study	Sample out of age range.
Collantoni (2020)	Cortical complexity in Anorexia Nervosa: A fractal dimension analysis	Sample out of age range.
Collantoni (2020)	Functional connectivity patterns and the role of 5-httlpr polymorphism on network architecture in female patients with anorexia nervosa	Sample out of age range.
Nickel (2019)	White matter abnormalities in the corpus callosum in acute and recovered anorexia nervosa patients-a diffusion tensor imaging study	Sample out of age range.
Van Opstal (2015)	Hypothalamic BOLD response to glucose intake and hypothalamic volume are similar in anorexia nervosa and healthy control subjects	Sample out of age range.
Yokokura (2019)	Alterations in serotonin transporter and body image-related cognition in anorexia nervosa	Sample out of age range.
Delvenne (1995)	Brain hypometabolism of glucose in anorexia nervosa: a PET scan study	Sample out of age range.
Coman (2013)	A disorder of a vulnerable self: Anorexia nervosa patients' understanding of disorder and self in the context of fMRI brain scanning	Sample out of age range.
Nagamitsu (2016)	Altered SPECT ^{123}I-iomazenil binding in the cingulate cortex of children with anorexia nervosa	Sample out of age range.
Lask (2005)	Functional Neuroimaging in Early-Onset Anorexia Nervosa	Sample out of age range.
Van Autreve (2016)	Differential Neural Correlates of Set-Shifting in the Bingeing-Purging and Restrictive Subtypes of Anorexia Nervosa: An fMRI Study	Sample out of age range.
Lai(2020)	Fractional amplitude of low frequency fluctuation in drug-naïve first-episode patients with anorexia nervosa: A resting-state fMRI study	Sample out of age range.
Lee (2014)	Resting-state synchrony between anterior cingulate cortex and precuneus relates to body shape concern in anorexia nervosa and bulimia nervosa.	Sample out of age range.
Schultz (2017)	Evidence for alterations of cortical folding in anorexia nervosa.	Sample rec-AN.
Fuglset (2016)	Brain volumes and regional cortical thickness in young females with anorexia nervosa.	Sample rec-AN.
Lambe (1985)	Cerebral Gray Matter Volume Deficits After Weight Recovery From Anorexia Nervosa	Sample rec-AN.
Frank (2002)	Reduced 5-HT2A Receptor Binding after Recovery from Anorexia Nervosa	Sample rec-AN.
Uher (2003)	Recovery and Chronicity in Anorexia Nervosa: Brain Activity Associated with Differential Outcomes	Sample rec-AN.
Frank (2005)	Increased dopamine D2/D3 receptor binding after recovery from anorexia nervosa measured by positron emission tomography and [11c]raclopride.	Sample rec-AN.
Wagner (2006)	Normal Brain Tissue Volumes after Long-Term Recovery in Anorexia and Bulimia Nervosa	Sample rec-AN.
Bailer (2007)	Serotonin transporter binding after recovery from eating disorders	Sample rec-AN.
Wagner (2007)	Altered Reward Processing in Women Recovered From Anorexia Nervosa	Sample rec-AN.
Mühlau (2007)	Gray Matter Decrease of the Anterior Cingulate Cortex in Anorexia Nervosa	Sample rec-AN.
Oberndorfer (2011)	Demand-specific alteration of medial prefrontal cortex response during an inhibition task in recovered anorexic women.	Sample rec-AN.

Joos (2011)	Grey matter deficit in long-term recovered anorexia nervosa patients.	Sample rec-AN.
Cowdrey (2011)	Increased neural processing of rewarding and aversive food stimuli in recovered anorexia nervosa.	Sample rec-AN.
Framton (2011)	Neurobiological Status at Initial Presentation Predicts Neuropsychological Functioning in Early Onset Anorexia Nervosa at Four-Year Follow Up	Sample rec-AN.
Cowdrey (2011)	Neural Responses to Emotional Faces in Women Recovered From Anorexia Nervosa	Sample rec-AN.
Yau (2013)	Alterations in White Matter Microstructure in Women Recovered From Anorexia Nervosa	Sample rec-AN.
Lázaro (2013)	Normal Gray and White Matter Volume After Weight Restoration in Adolescents With Anorexia Nervosa	Sample rec-AN.
Boehm (2016)	Partially Restored Resting-State Functional Connectivity in Women Recovered From Anorexia Nervosa	Sample rec-AN.
Bang (2016)	Normal gray matter volumes in women recovered from anorexia nervosa: a voxel-based morphometry study.	Sample rec-AN.
Bang (2018)	Normal white matter microstructure in women long-term recovered from anorexia nervosa: A diffusion tensor imaging study.	Sample rec-AN.
Geisler (2019)	Altered global brain network topology as a trait marker in patients with anorexia nervosa.	Sample rec-AN.
Kornreich (1991)	CT and MR Evaluation of the Brain in Patients with Anorexia Nervosa	Low score in NewCastle-Ottawa scale.

Supplemental Table S2a. Evaluation of the eligible studies with Newcastle-Ottawa scale- **Case-Control studies.**

Study	*Selection*				*Comparability*		*Exposure*			*Total*
	**Case definition**	**Representativeness of the of the cases**	**Selection of controls**	**Definition of controls**	**Comparability on age**	**Comparability on other risk factors**	**Ascertainment of exposure**	**Same method for cases and controls**	**Non-response rate**	
Blasel (2011)	1	1	0	0	1	1	1	1	1	7
Castro-Fornieles (2007)	1	1	1	0	1	0	1	1	1	7
Schlemmer (1997)	1	0	1	1	1	0	1	1	1	7
King (2015)	1	0	1	1	1	1	1	1	1	8
Myrvang (2018)	1	1	1	1	1	1	1	1	1	9
Yue (2018)	1	0	1	1	1	1	1	1	1	8
Olivo (2018)	1	1	1	1	1	1	1	1	1	9
Fujisawa (2015)	1	1	1	1	1	1	1	1	1	9
Golden (1996)	1	1	0	1	0	1	1	1	1	7
Monzon (2017)	1	0	1	1	1	1	1	1	1	8
Akgül (2016)	1	0	0	1	1	1	1	1	1	7
Katzman (1996)	1	1	1	0	0	1	1	1	1	7
Neumärker (2000)	1	0	1	1	1	1	1	0	1	7
Castro-Fornieles (2009)	1	1	1	1	1	1	1	1	1	9
Takano (2001)	1	1	1	1	0	0	1	1	1	7
Kojima (2005)	1	1	1	0	0	0	1	1	1	6
Travis (2015)	1	1	1	1	1	0	1	1	1	8
Cha (2016)	1	1	0	1	1	1	1	1	1	8
Hu (2017)	1	1	1	1	1	1	1	1	1	9
Gaudio (2017)	1	1	1	1	1	0	1	1	1	8
Pfuhl (2016)	1	1	1	1	1	0	1	1	1	8
von Schwanenflug (2018)	1	0	0	1	1	0	1	1	1	7
Gaudio (2015)	1	1	1	1	1	0	1	1	1	8
Seidel (2019)	1	1	0	1	1	0	1	1	1	7

Amianto (2013)	1	1	1	1	1	1	1	1	1	9
Geisler (2015)	1	1	1	1	1	0	1	1	1	8
Bohem (2014)	1	1	1	1	1	0	1	1	1	8
Gaudio (2018)	1	1	1	1	1	0	1	1	1	8
Uniacke (2018)	1	1	0	1	1	1	1	1	1	8
Vogel (2015)	1	1	1	1	1	1	1	1	1	9
Olivo (2019)	1	1	1	1	1	0	1	1	1	8

Supplemental Table S2b. Evaluation of the eligible studies with Newcastle-Ottawa scale- **Cohort studies.**

Study	*Selection*				*Comparability*		*Exposure*			*Total*
	Representativeness	*Selection of non-exposed*	*Ascertainment of exposure*	*Outcome not present at start*	*On age*	*On other risk factors*	*Assessment of outcome*	*Long enough follow up (12months)*	*Adequacy of follow-up*	
MRI										
Katzman (1997)	1	1	1	1	1	1	1	0	1	8
Neumärker (2000)	1	1	1	1	1	1	1	0	1	8
Castro-Fornieles (2009)	1	1	1	1	1	1	1	0	1	8
Akgül (2016)	1	1	1	1	1	1	1	1	1	9
Bernardoni (2018)	1	1	1	1	1	1	1	0	1	8
Golden (1996)	1	1	1	1	1	1	1	0	1	8
Monzon (2017)	1	1	1	1	1	1	1	0	1	8
MRI-DTI										
Vogel (2016)	1	1	1	1	1	1	1	0	1	8
von Schwanenflug (2018)	1	1	1	1	1	1	1	0	1	8
MRS										
Castro-Fornieles (2007)	1	1	1	1	1	1	1	0	1	8
SPECT										
Matsumoto (2006)	1	1	1	1	1	1	1	0	1	8
Kojima (2005)	1	1	1	1	1	1	1	0	1	8
Komatsu (2010)	1	1	1	1	1	1	1	0	1	8

PRISMA 2009 Checklist

Section/topic	#	Checklist item	Reported on page #
**TITLE**			
Title	1	Identify the report as a literature review.	1
**ABSTRACT**			
Structured summary	2	Provide a structured summary including, as applicable: background; objectives; data sources; study eligibility criteria, participants, and interventions; study appraisal and synthesis methods; results; limitations; conclusions and implications of key findings;	1
**INTRODUCTION**			
Rationale	3	Describe the rationale for the review in the context of what is already known about your topic.	2-3
Objectives	4	Provide an explicit statement of questions being addressed with reference to participants, interventions, comparisons, outcomes, and study design (PICOS).	
**METHODS**			
Eligibility criteria	5	Specify study characteristics (e.g., PICOS, length of follow-up) and report characteristics (e.g., years considered, language, publication status) used as criteria for eligibility, giving rationale.	3-4
Information sources	6	Describe all information sources (e.g., databases with dates of coverage) in the search and date last searched.	3-4
Search	7	Present full electronic search strategy for at least one database, including any limits used, such that it could be repeated.	3-4
Study selection	8	State the process for selecting studies (i.e., screening, eligibility).	3-4
Risk of bias in individual studies	9	Describe methods used for assessing risk of bias of individual studies (including specification of whether this was done at the study or outcome level).	4
Risk of bias across studies	10	Specify any assessment of risk of bias that may affect the cumulative evidence (e.g., publication bias, selective reporting within studies).	4

Section/topic	#	Checklist item	Reported on page #
**RESULTS**			
Study selection	11	Give numbers of studies screened, assessed for eligibility, and included in the review, with reasons for exclusions at each stage, ideally with a flow diagram.	4
Study characteristics	12	For each study, present characteristics for which data were extracted (e.g., study size, PICOS, follow-up period) and provide the citations.	5
Synthesis of results of individual studies	13	For all outcomes considered (benefits or harms), present, for each study: (a) summary of results and (b) relationship to other studies under review (e.g. agreements or disagreements in methods, sampling, data collection or findings).	5-25
**DISCUSSION**			
Summary of evidence	14	Summarize the main findings including the strength of evidence for each main outcome; consider their relevance to key groups (e.g., healthcare providers, users, and policy makers).	26-32
Limitations	15	Discuss limitations at study and outcome level (e.g., risk of bias), and at review-level (e.g., incomplete retrieval of identified research, reporting bias).	33
**CONCLUSION**			
Conclusions	16	Provide a general interpretation of the results in the context of other evidence, and implications for future research.	34

Adapted from: Moher D, Liberati A, Tetzlaff J, Altman DG, The PRISMA Group (2009). Preferred Reporting Items for Systematic Reviews and Meta-Analyses: The PRISMA statement. *PLoS Medicine*, 6(6), e1000097. doi:10.1371/journal.pmed1000097

For more information, visit: **www.prisma-statement.org**.
